# Origin and evolution of the octoploid strawberry genome

**DOI:** 10.1038/s41588-019-0356-4

**Published:** 2019-02-25

**Authors:** Patrick P. Edger, Thomas J. Poorten, Robert VanBuren, Michael A. Hardigan, Marivi Colle, Michael R. McKain, Ronald D. Smith, Scott J. Teresi, Andrew D. L. Nelson, Ching Man Wai, Elizabeth I. Alger, Kevin A. Bird, Alan E. Yocca, Nathan Pumplin, Shujun Ou, Gil Ben-Zvi, Avital Brodt, Kobi Baruch, Thomas Swale, Lily Shiue, Charlotte B. Acharya, Glenn S. Cole, Jeffrey P. Mower, Kevin L. Childs, Ning Jiang, Eric Lyons, Michael Freeling, Joshua R. Puzey, Steven J. Knapp

**Affiliations:** 1 Department of Horticulture, Michigan State University, East Lansing, MI USA; 2 Ecology, Evolutionary Biology and Behavior, Michigan State University, East Lansing, MI USA; 3 Department of Plant Sciences, University of California–Davis, Davis, California, USA; 4 Plant Resilience Institute, Michigan State University, East Lansing, MI USA; 5 Department of Biological Sciences, University of Alabama, Tuscaloosa, AL USA; 6 Department of Biology, College of William and Mary, Williamsburg, VA USA; 7 School of Plant Sciences, University of Arizona, Tucson, AZ USA; 8 NRGene, Ness Ziona, Israel; 9 Dovetail Genomics, Santa Cruz, CA USA; 10 Center for Plant Science Innovation, University of Nebraska, Lincoln, NE USA; 11 Department of Plant Biology, Michigan State University, East Lansing, MI USA; 12 Center for Genomics Enabled Plant Science, Michigan State University, East Lansing, MI USA; 13 Department of Plant and Microbial Biology, University of California, Berkeley, Berkeley, CA USA

**Keywords:** Transcriptomics, Plant hybridization, Gene expression

## Abstract

Cultivated strawberry emerged from the hybridization of two wild octoploid species, both descendants from the merger of four diploid progenitor species into a single nucleus more than 1 million years ago. Here we report a near-complete chromosome-scale assembly for cultivated octoploid strawberry (*Fragaria* *×* *ananassa*) and uncovered the origin and evolutionary processes that shaped this complex allopolyploid. We identified the extant relatives of each diploid progenitor species and provide support for the North American origin of octoploid strawberry. We examined the dynamics among the four subgenomes in octoploid strawberry and uncovered the presence of a single dominant subgenome with significantly greater gene content, gene expression abundance, and biased exchanges between homoeologous chromosomes, as compared with the other subgenomes. Pathway analysis showed that certain metabolomic and disease-resistance traits are largely controlled by the dominant subgenome. These findings and the reference genome should serve as a powerful platform for future evolutionary studies and enable molecular breeding in strawberry.

## Abstract (web summary)

Chromosome-scale assembly for the cultivated octoploid strawberry (*Fragaria* × *ananassa*) uncovers the origin and evolutionary processes that shaped this complex allopolyploid, providing a useful resource for genome-wide analyses and molecular breeding.

## Main

The cultivated garden strawberry (*Fragaria* × *ananassa*), an allo-octoploid (2*n* = 8*x* *=* 56), has a unique natural and domestication history, originating as an interspecific hybrid between wild octoploid progenitor species approximately 300 years before present^1^. The genomes of the progenitor species, *Fragaria virginiana* and *Fragaria chiloensis*, are the products of polyploid evolution: they were formed by the fusion of and interactions among genomes from four diploid progenitor species (that is, subgenomes) approximately 1 million years before present^2^. Whereas two of the diploid progenitor species have been identified^3^, the other two diploid progenitor species have remained unknown. Moreover, the history of events leading to the formation of the octoploid lineage and the evolutionary dynamics among the four subgenomes that restabilized cellular processes after ‘genomic shock’^4^ in allopolyploids remain poorly understood. Here, we present what is, to our knowledge, the first chromosome-scale assembly of an octoploid strawberry genome, the identities of the extant diploid progenitor species of each subgenome, and novel insights into the collective evolutionary processes involved in establishing a dominant subgenome in this highly polyploid species.

The Rosaceae are a large eudicot family including a rich diversity of crops with major economic importance worldwide, such as nuts (for example, almonds), ornamentals (for example, roses), pome fruits (for example, apples), stone fruits (for example, peaches), and berries (for example, strawberries)^5^. Strawberries are prized by consumers, largely because of their complex array of flavors and aromas. The genus *Fragaria* was named by the botanist Carl Linnaeus, on the basis of the Latin word ‘fragrans’, meaning ‘sweet scented’, describing its striking, highly aromatic fruit^6^. A total of 22 wild species of *Fragaria* have been described, ranging from diploid (2*n* = 2*x* *=* 14) to decaploid (2*n* = 10*x* *=* 70)^7^. The genus *Fragaria* is highly interfertile between and within ploidy levels, thus leading to the natural formation of higher-polyploid species^8,9^.

Polyploid events, also known as whole-genome duplications, have been an important recurrent process throughout the evolutionary history of eukaryotes and have probably contributed to novel and varied phenotypes^10–13^. Polyploids are grouped into two main categories: autopolyploids and allopolyploids, involving either a single or multiple diploid progenitor species, respectively^14,15^. Many crop species are allopolyploids^16^, thus contributing to the emergence of important agronomic traits such as spinnable fibers in cotton^17^, diversified morphotypes in *Brassica*^18^, and varied aroma and flavor profiles in strawberry^19^. Allopolyploids face the challenge of organizing distinct parental subgenomes—each with a unique genetic and epigenetic makeup shaped by independent evolutionary histories—residing within a single nucleus^15^. Previous studies have proposed, as part of the ‘subgenome dominance’ hypothesis^20^, that the establishment of a single dominant subgenome may resolve various (epi)genetic conflicts in allopolyploids^21–24^. However, understanding of the underlying mechanisms and ultimate consequences of subgenome dominance remains largely incomplete^25^.

Subgenome-level analyses in most allopolyploid systems are greatly hindered by the inability to confidently assign parental gene copies (that is, homoeologs) to each subgenome, owing to both large-scale chromosomal changes and homoeologous exchanges that shuffle and replace homoeologs among parental chromosomes^26–29^. Octoploid strawberry still has a complete set of homoeologous chromosomes from all four parental subgenomes, thus greatly simplifying homoeolog assignment. Furthermore, gene sequences from extant relatives of the diploid progenitor species, which probably still exist for octoploid strawberry^3^, can be used to accurately assign homoeologs to each parental subgenome^29^. However, a high-quality reference genome for the octoploid is needed to fully exploit strawberry as a model system for studying allopolyploidy as well as to provide a platform for identifying biologically and agriculturally important genes and applying genomic-enabled breeding approaches^30^. The assembly of the octoploid strawberry genome, with an estimated genome size of 813.4 Mb, has been particularly challenging because of its high heterozygosity and ploidy level^31^. For example, the most recently published version of the octoploid strawberry genome is highly fragmented, with more than 625,000 scaffolds, and largely incomplete, with less than 660 Mb assembled after removal of the numerous gaps^31^. Thus, that version of the genome, owing to its overall highly fragmented nature, has not been a useful resource for genome-wide analyses including the discovery of molecular markers for breeding.

## Results

### Assembly and annotation of the octoploid strawberry genome

**Fig. 1:**
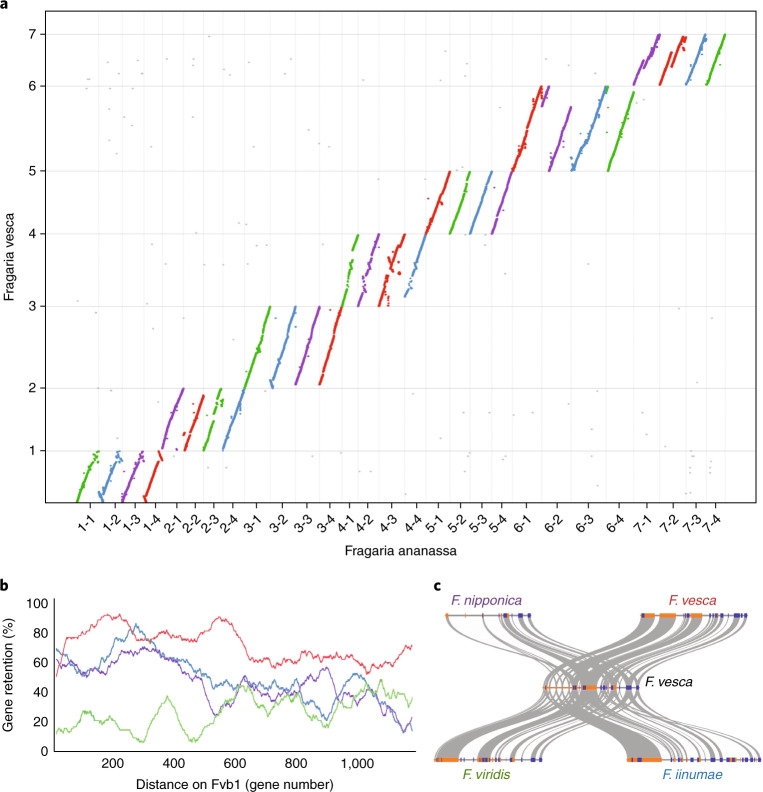
Collinearity of the diploid and octoploid strawberry genomes. **a**, Macrosyntenic comparison of the entire *Fragaria* × *ananassa* and diploid *F. vesca*^37^ genomes, with each homoeologous chromosome set colored according to its diploid progenitor species (*F. vesca* in red, *F. nipponica* in purple, *F. iinumae* in blue, and *F. viridis* in green). Details are provided in Supplementary Table 8. *F. vesca* and *F. ananassa* chromosomes are shown on the *y* axis and *x* axis, respectively. **b**, Gene-retention patterns among the four homoeologous copies of chromosome 1, with color coding as in **a**. The relative distance along the *F. vesca* chromosome is shown on the *x* axis with the total number of analyzed genes. The percentage of genes retained is shown on the *y* axis, as estimated with sliding windows of 100 genes. The chromosomes of *F. vesca*^37^ are named Fvb1 through Fvb7. **c**, A microsyntenic comparison of a region on chromosome 1 between diploid *F. vesca* and the four homoeologous regions in *Fragaria* × *ananassa*. Gray lines indicate shared syntenic gene pairs, and relative orientation is shown in blue (forward) or orange (reverse). The four subgenomes of *Fragaria* × *ananassa* are labeled with corresponding diploid species names of potential origins.

Our goal was to obtain a high-quality reference genome for the *Fragaria* × *ananassa* cultivar ‘Camarosa’, one of the most historically important and widely grown strawberry cultivars worldwide. We sequenced the genome through a combination of short- and long-read approaches, including Illumina, 10X Genomics, and PacBio, totaling 615-fold coverage of the genome (Supplementary Table 1). Illumina (455-fold coverage) and 10X Genomics (117-fold coverage) data were assembled and scaffolded with the software package DenovoMAGIC3 (NRGene) (Supplementary Table 2), which has recently been used to assemble the allotetraploid wheat (*Triticum turgidum*) genome^32^. We further scaffolded the genome to chromosome scale by using Hi-C data (401-fold coverage) in combination with the HiRise pipeline (Dovetail) (Supplementary Figs. 1–3), then performed gap-filling with 43-fold-coverage error-corrected PacBio reads with PBJelly^33^ (Supplementary Table 3). The total length of the final assembly is 805,488,706 bp, distributed across 28 chromosome-level pseudomolecules (Fig. 1) and representing ~99% of the estimated genome size, on the basis of flow cytometry measurements. A genetic map for *Fragaria* *×* *ananassa*^34^ was used to correct any misassemblies, and comparisons to *Fragaria vesca* were used to identify homoeologous chromosomes.

We annotated 108,087 protein-coding genes along with 30,703 genes encoding long noncoding RNAs (lncRNAs), which were subdivided into 15,621 long intergenic noncoding RNAs, 9,265 antisense overlapping transcripts (AOT-lncRNAs), and 5,817 sense overlapping transcripts (SOT-lncRNAs) (Supplementary Table 4). Gene annotation and genome-assembly quality were evaluated with the Benchmarking Universal Single-Copy Orthologs v 2 (BUSCO)^35^ method (Supplementary Table 5). Most (99.17%) of the 1,440 core genes in the embryophyta dataset were identified in the annotation, thus supporting a high-quality genome assembly. The repetitive components of the nuclear genome were annotated with a custom-repeat-library approach^36^, including DNA transposons, long-terminal-repeat retrotransposons (LTR-RTs; for example, *Copia* and *Gypsy*), and non-LTR retrotransposons (Supplementary Table 6 and Supplementary Fig. 4). Transposable element (TE)-related sequences make up ~36% of the total genome assembly, and LTR-RTs are the most abundant TEs (~28%). The plastid and mitochondrial genomes were also assembled, annotated, and verified for completeness (Supplementary Fig. 5).

### Origin of octoploid strawberry

**Fig. 2:**
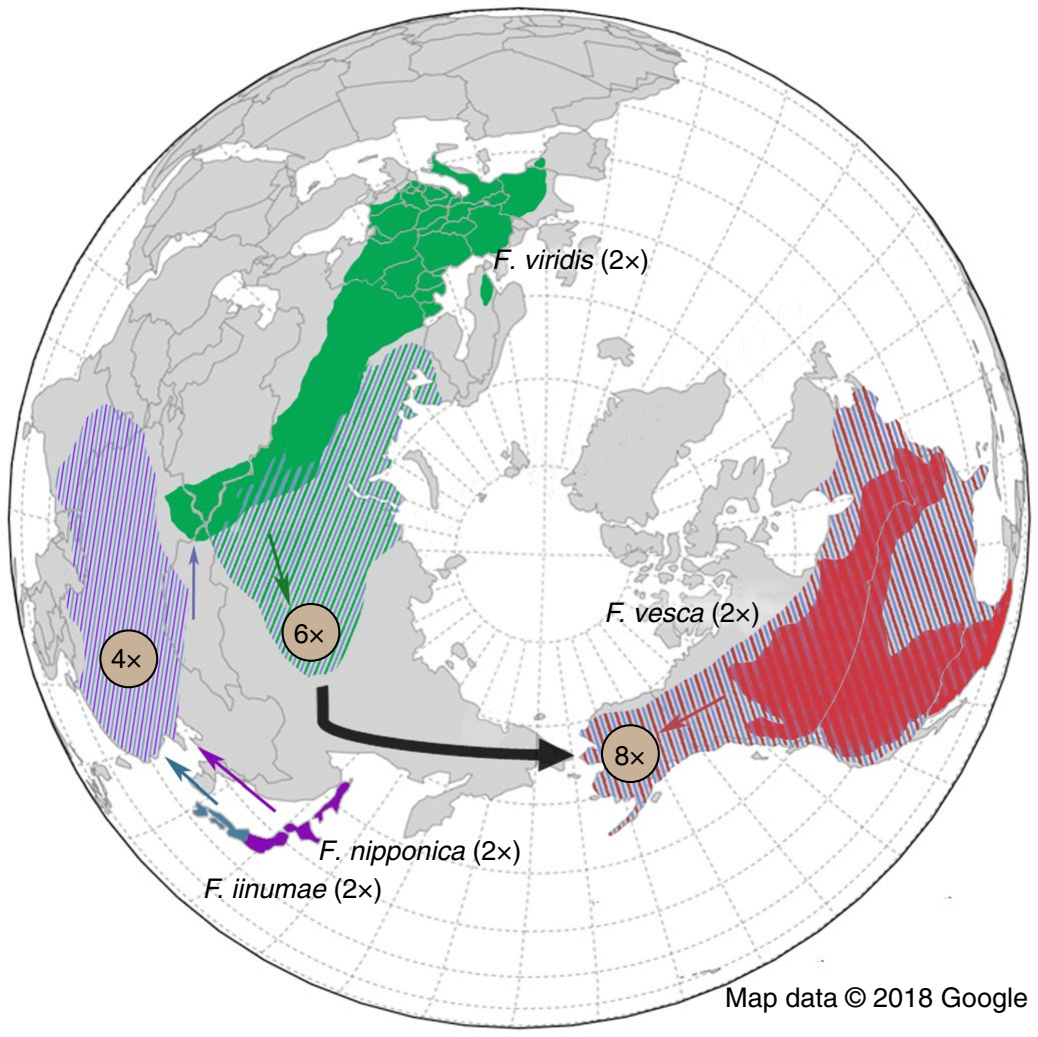
The evolutionary history of the octoploid strawberry. North-polar projection of present day. Geographic distributions of extant relatives of the diploid (2×) progenitors of *Fragaria* × *ananassa*, the putative intermediate tetraploid (4×) and hexaploid (6×) progenitors of *Fragaria* × *ananassa*, and extant wild octoploid (8×) species in North America. The colors associated with each diploid progenitor are as in Fig. 1. Map data were obtained from Google Maps (see URLs).

Using the *Fragaria* *×* *ananassa* reference-genome assembly, we sought to identify the extant diploid relatives of each subgenome donor^37^. Previous phylogenetic studies aimed at identifying these progenitor species, often analyzing a limited number or different sets of molecular markers, have obtained inconsistent results^3,38,39^. However, *F. vesca* has long been suspected to be a progenitor, on the basis of meiotic chromosome pairing^40^; subsequent molecular phylogenetic analyses supported it being one of the diploid progenitors along with *Fragaria iinumae* and two additional unknown species^3^. We sequenced and de novo assembled 31 transcriptomes of every described diploid *Fragaria* species, which we used to identify progenitor species on the basis of the phylogenetic analysis of 19,302 nuclear genes in the genome (Fig. 2, Supplementary Figs. 6–8 and Supplementary Table 7). To our knowledge, this is the most comprehensive molecular phylogenetic analysis of the genus *Fragaria* to date, including the greatest number of molecular markers and sampling of diploid species, aimed at identifying the extant relatives of the progenitor species of octoploid strawberry (Supplementary Fig. 9 and Supplementary Table 8).

Our phylogenetic analyses provided strong genome-wide support for the two diploid progenitor species that had been previously hypothesized and identified the two previously unknown diploid progenitors. This discovery, together with the geographic distributions, natural history, and genomic footprints of the diploid species, provided a model for the chronological formation of intermediate polyploids that culminated in the formation of the octoploid (Fig. 2). Our phylogenetic analyses revealed *F. iinumae* and *Fragaria nipponica* as two of the four extant diploid progenitor species, both of which are endemic to Japan and in geographic proximity to all five described tetraploid species in China. The third species identified in our analyses, *Fragaria viridis*, is geographically distributed in Europe and Asia, and partially overlaps with the sole hexaploid species, *Fragaria moschata*. Therefore, we hypothesized that these tetraploid and hexaploid species may be evolutionary intermediates between the diploids and the wild octoploid species. This possibility is supported by a previous phylogenetic analysis identifying *F. viridis* as a possible parental contributor to both *F. moschata* and the octoploid event^41^. Finally, we identified *F. vesca* subsp. *bracheata*, which is endemic to the western part of North America, spanning Mexico to British Columbia, as the fourth parental contributor. Our species sampling also included two other *F. vesca* subspecies: *F. vesca* subsp. *vesca*, which is distributed from Europe to the Russian Far East, and *F. vesca* subsp. *californica*, which is endemic to the coast of California.

Octoploid strawberry species are geographically restricted to the New World and are largely distributed across North America, with the exception of isolated *F. chiloensis* populations in Chile and the Hawaiian Islands^42^. Therefore, our phylogenetic analyses combined with the geographic distributions of extant species not only support a North American origin for the octoploid strawberry but also suggest that *F. vesca* subsp. *bracheata* was probably the last diploid progenitor species to contribute to the formation of the ancestral octoploid strawberry. This possibility is further supported by a previous study revealing *F. vesca* subsp. *bracheata* as the likely maternal donor of the octoploid event, on the basis of the phylogenetic history of the plastid genome^2^. This finding is consistent with our analysis of the plastid genome of ‘Camarosa’ (Supplementary Fig. 10). Thus, these data suggest that the hexaploid ancestor probably crossed into North America from Asia and hybridized with native populations of *F. vesca* subsp. *bracheata*, an event dated at ~1.1 million years before present^2^. Our phylogenetic analysis also identified related diploid species possibly arising from ancient hybridization and introgression events with putative progenitor species or issues related to incomplete lineage sorting and/or missing data (Supplementary Fig. 6). Future studies will be able to more thoroughly investigate these possibilities after reference quality genomes are assembled for these other diploid progenitor species.

### Subgenome dominance in allopolyploids

After most ancient allopolyploid events, one of the subgenomes, commonly referred to as the ‘dominant’ subgenome, emerges with significantly greater gene content and more highly expressed homoeologs (that is, postpolyploidy duplicate genes) than those of the other ‘submissive’ subgenome(s)^21^. Biased fractionation, which results in greater gene content of the dominant subgenome^43^, was first described in the model plant *Arabidopsis thaliana*^21^ and later described in *Zea mays* (maize)^20^, *Brassica rapa* (Chinese cabbage)^44^, and *Triticum aestivum* (bread wheat)^45^. The dominant subgenome has also been shown to be under stronger selective constraints^46–48^ and to be heritable through successive allopolyploid events^49^, and, as predicted^22^, it is not observed in ancient autopolyploids^50–52^. Moreover, subgenome expression dominance has recently been shown to occur instantly after interspecific hybridization and to increase over successive generations in monkeyflower^23^. However, some allopolyploids, including *Capsella bursa-pastoris*^53^ and *Cucurbita* species^54^, do not exhibit subgenome dominance.

The emergence of a dominant subgenome may resolve various genetic and epigenetic conflicts that arise from the genomic merger of divergent diploid progenitor species^4,55^, including mismatches between transcriptional regulators and their target genes^24^. The mechanistic basis of subgenome dominance, at least in part, appears to be related to subgenome differences in the content and regulation of TEs^22,56^. Gene expression levels are negatively correlated with the density of nearby TEs^56^ (Supplementary Fig. 11). Thus, the merger of subgenomes with different TE densities results in higher gene expression for the dominant homoeolog with fewer TEs^22^. The abundance and distribution of TEs can be used to predict gene expression dominance and eventual gene loss at the individual homoeolog level^23^.

Having identified the extant diploid relatives of octoploid strawberry, we used this information to investigate the evolutionary dynamics among the four subgenomes. We identified a dominant subgenome that was contributed by the *F. vesca* progenitor (Fig. 1) and has retained 20.2% more protein-coding genes and 14.2% more lncRNA genes, and has overall 19.5% fewer TEs than the other homoeologous chromosomes (Supplementary Table 9). The overall TE densities near genes were also lowest for *F. vesca* compared with the other parental subgenomes (Supplementary Fig. 11). Furthermore, we identified ~40.6% more tandem gene duplications on homoeologous chromosomes of *F. vesca* compared with the other subgenomes (Supplementary Table 9). The *F. vesca* subgenome, compared with the other subgenomes, also contains a greater number of tandem gene arrays as well as larger average tandem-gene-array sizes on six of seven homoeologous chromosomes. These findings suggest that the dominant *F. vesca* subgenome, compared with the other three subgenomes, has been under stronger selective constraints to retain genes, including tandemly duplicated genes known to be biased toward gene families that encode important adaptive traits^57,58^. For example, major disease-resistance genes in plants, including nucleotide-binding-site leucine-rich-repeat genes (NBS-LRRs), which are usually clustered in tandem arrays^59^, are biased toward the dominant *F. vesca* subgenome (*χ*2 test, *P* < 0.0001; Supplementary Fig. 12).

Because strawberry production is threatened by several agriculturally important diseases, we analyzed, in greater depth, the major family of plant resistance (R) genes^60,61^. Collectively, 423 NBS-LRR genes were identified, including 195 encoding an N-terminal coiled-coil (CC), 79 encoding toll interleukin 1 receptor (TIR), and 24 encoding resistance to powdery mildew 8 (RPW8) domains (Supplementary Fig. 12). Recent work has demonstrated that many R proteins recognize pathogen effectors through integrated decoy domains^62^, and the *F. vesca* genome encodes 20 such protein models^63^. *Fragaria* *×* *ananassa* has a greatly expanded set of 105 diverse domains that are fused to the R-protein structures and have the potential to function as integrated decoys^62^ (Supplementary Fig. 13 and Supplementary Dataset 1). Only a few resistance genes have been phenotypically identified in *Fragaria* × *ananassa*, but none have been functionally characterized^64–66^. The annotated genome thus provides a framework for accelerating R-gene discovery, connecting phenotype to genotype, and pyramiding R genes by developing targeted, homoeolog-specific molecular markers.

Although chromosomes contributed by the *F. vesca* progenitor retained the most genes overall, certain regions on chromosomes from the other progenitor species retained higher numbers of ancestral genes (Fig. 1b and Supplementary Fig. 14). Further analysis revealed that these regions are the products of homoeologous exchanges (HEs) or gene-conversion events^28,67,68^ (Supplementary Figs. 15 and 16). Notably, most HEs in octoploid strawberry involved replacements of the submissive homoeologs by corresponding regions of the dominant *F. vesca* subgenome (Supplementary Table 10). For example, our phylogenetic and comparative genomic analyses showed that HEs are 7.3× biased toward the *F. vesca* subgenome compared with *F. iinumae*, but they are not unidirectional as previously reported^3^. HEs were even more biased toward the *F. vesca* subgenomes compared with the other two subgenomes (9.8× for *F. viridis* and 10.4× for *F. nipponica*). These analyses validate findings from a previous study in wild octoploid strawberry^3^ and show that portions of the *F. iinumae* subgenome have been replaced with the *F. vesca* subgenome (Fig. 1b). Here, we identified HEs ranging in size from single genes to megabase-sized regions on chromosomes (Supplementary Table 10), findings similar to the patterns observed in other allopolyploids including *Brassica napus* (rapeseed)^27,28^, *Gossypium hirsutum* (cotton)^67,69^, and bread wheat^70^. The observed bias of HEs genome wide may be due to selection favoring the maintenance of proper network stoichiometry^71^ and altered dosage of certain gene products^72^ during the establishment of the dominant subgenome. Interestingly, 32.6% of NBS-LRR genes encoded on the three submissive subgenomes are derived from HE with the *F. vesca* subgenome. This result suggests that although the *F. vesca* subgenome may also dominate disease resistance in strawberry, the maintained diversity of resistance mechanisms contributed by the other three diploid progenitors may also have been under selection.

**Fig. 3:**
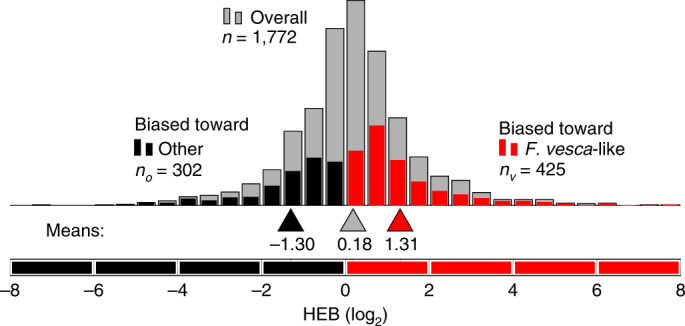
Subgenome expression dominance. Homoeolog expression bias (HEB) for all testable homoeolog pairs, shown in gray histograms. Testable homoeolog pairs (*n*) are those that could confidently be identified as homoeologous on the basis of synteny and assigned to a subgenome with phylogenetic support (>80% bootstrap), and that had at least one read in each transcriptome dataset. Homoeolog pairs significantly biased toward the *F. vesca* homoeolog are shown in red, and pairs significantly biased toward the ‘other’ homoeolog from one of the other three diploid progenitors are shown in black.

Finally, we examined gene expression in diverse organs to test whether the dominant *F. vesca* subgenome is more highly expressed than the submissive genomes (Fig. 3), as predicted by the subgenome-dominance hypothesis^22,25^. The density of TEs near genes was found to be negatively correlated with gene expression across all subgenomes (Supplementary Fig. 11a). Because HEs reshuffled and replaced homoeologs across each of the four parental chromosomes, only homoeolog pairs that had support for subgenome assignment were evaluated for subgenome expression dominance (that is, homoeolog expression bias). Our analyses revealed that the dominant *F. vesca* subgenome, which had the lowest overall TE densities near genes of all subgenomes (Supplementary Fig. 11b; Kolmogorov–Smirnov test, *P* < 10^−33^), encodes more significantly dominantly expressed homoeologs than the other three submissive subgenomes combined (Fig. 3c). This finding supports the hypothesis that subgenome expression dominance is influenced by overall TE-density differences between subgenomes^22^. At the individual homoeolog level, many dominantly expressed homoeologs were also contributed by one of the three submissive subgenomes. This observation was expected, given the variation in TE densities near homoeologs in each of the diploid progenitor genomes^23,73^.

Most HEs in octoploid strawberry resulted in the dominant *F. vesca* subgenome replacing the corresponding homoeologous regions of one of the submissive subgenomes. Thus, the observed homoeolog expression bias toward the *F. vesca* subgenome in Fig. 3 is an underestimate of transcriptome-wide expression dominance (68.7% of all transcripts). This bias has resulted in certain biological pathways being largely controlled by a single dominant subgenome. Our analyses revealed that certain metabolic pathways, including those that give rise to strawberry flavor, color, and aroma, are largely controlled by the dominant subgenome. For example *F. vesca* homoeologs in octoploid strawberry are responsible for 88.8% of the biosynthesis of anthocyanins, the metabolites responsible for the red pigments in ripening strawberry fruit; 89.2% of the biosynthesis of geranyl acetate, a terpene associated with fruit aroma; and 95.3% of the biosynthesis of fructose associated with sweetness (Supplementary Dataset 2). Similar results have been found in allotetraploid *Brassica juncea*, in which many dominant homoeologs have been found to be related to glucosinolate biosynthesis and to show signs of positive selection^74^.

## Discussion

We present what is, to our knowledge, the first chromosome-scale genome assembly for an octoploid strawberry—the highest-level polyploid genome of this quality assembled to date. Analysis of this genome allowed us to identify each of the diploid progenitor species, reconstruct the evolutionary history of the octoploid event, and investigate the evolution of a dominant subgenome. Our data support the hypothesis that subgenome dominance in an allopolyploid is established by TE-density differences near homoeologous genes in each of the diploid progenitor genomes^22^. Furthermore, our results show that the *F. vesca* subgenome has increased in dominance over time by having retained significantly more ancestral genes and a greater number of tandemly duplicated genes than the other three subgenomes, and replaced large portions of the submissive subgenomes via homoeologous exchanges. These trends, combined with subgenome expression dominance, have resulted in many traits being largely controlled by a single dominant subgenome in octoploid strawberry. This finding is consistent with results from a recent report indicating that the dominant subgenome in maize contributes more to phenotypic variation than the submissive subgenome^48^. This reference genome should serve as a powerful platform for breeders to develop homoeolog-specific markers to track and leverage allelic diversity at target loci. Thus, we anticipate that this new reference genome, combined with insights into subgenome dominance, will greatly accelerate molecular breeding efforts in the cultivated garden strawberry.

### URLs

Sequence Read Archive, https://www.ncbi.nlm.nih.gov/sra/; Dryad, 10.5061/dryad.b2c58pc; PhyDS, https://github.com/mrmckain/PhyDS/; GDR, https://www.rosaceae.org/; CoGe, https://genomevolution.org/r/tx72/; RefTrans, https://github.com/mrmckain/RefTrans/; annoBTD, https://github.com/mrmckain/annoBTD/; Mitofy, http://dogma.ccbb.utexas.edu/mitofy/; dotPlotly, https://github.com/tpoorten/dotPlotly/; NCBI Conserved Domain Database, www.ncbi.nlm.nih.gov/Structure/bwrpsb/bwrpsb.cgi/; Pfam database, www.ebi.ac.uk/Tools/pfa/pfamscan/; FastQC, https://www.bioinformatics.babraham.ac.uk/projects/fastqc/; R, https://www.r-project.org/; Repeat-Masker, http://www.repeatmasker.org/; RepeatModeler, http://www.repeatmasker.org/RepeatModeler/; Google Maps, https://www.google.com/maps/.

## Methods

### Plant material

The cultivar ‘Camarosa’ was selected because of its importance to the industry; historically, it has been one of the most widely grown short-day varieties worldwide, and it remains an important genotype in breeding programs. The haploid genome size (~813.4 Mb) was estimated through flow cytometry with four technical replicates at the Flow Cytometry Core at Benaroya Research Institute at Virginia Mason (Supplementary Dataset 3).

### Genomic sequencing

High-molecular-weight genomic DNA was isolated from young leaf tissue, after a 72-h dark treatment, through a modified nuclei-preparation method^75,76^, and the quality was verified through pulsed-field gel electrophoresis. A total of five PacBio 20-kb libraries were generated with a SMRTbell Template Prep Kit (PacBio) and were sequenced with 67 SMRT cells on the PacBio RSII platform at the UC Davis DNA Sequencing Facility. A total of 67 Gb (~82.4×) of PacBio sequence data was generated with an *N*50 read length of 17,699 bp (Supplementary Table 3). DNA fragments longer than 50 kb were used to construct a 10X Gemcode library with a Chromium instrument (10X Genomics) and sequenced on a HiSeqX system (Ilumina) with paired-end, 150-bp reads at the HudsonAlpha Institute for Biotechnology. A total of ~95 Gb (~117× fold coverage) of 10X Chromium library data was sequenced (Supplementary Table 1). Finally, five size-selected Illumina genomic libraries ranging from 470 bp to 10 kb were constructed (Supplementary Table 1). The ~470-bp and ~800-bp libraries were made with a Illumina TruSeq DNA PCR-free Sample Preparation V2 Kit. The two ~470-bp libraries were designed to produce ‘overlapping libraries’ after sequencing with paired-end, 265-bp reads on an Illumina Hiseq2500 system, producing ‘stitched’ reads of approximately 265 bp to 520 bp in length. To increase sequence diversity and depth, we constructed three separate mate-pair (MP) libraries with jumps of 2–5 kb, 5–7 kb, and 7–10 kb, with an Illumina Nextera Mate-Pair Sample Preparation Kit. The 800-bp library was sequenced on an Illumina HiSeq2500 system with paired-end, 160-bp reads, and the MP libraries were sequenced on an Illumina HiSeq4000 system with paired-end, 150-bp reads. A total of ~370 Gb (~455× fold coverage) of additional Illumina sequencing data was generated (Supplementary Table 1). Illumina library construction and sequencing were conducted at the Roy J. Carver Biotechnology Center, University of Illinois at Urbana-Champaign.

### Genome assembly

The genome was assembled with the DeNovoMAGIC software platform (NRGene), a DeBruijn-graph-based assembler designed for higher polyploid, heterozygous and/or repetitive genomes^32,77^. The Chromium 10X data were used to phase haplotypes and support scaffold validation and further elongation of the phased scaffolds. Dovetail HiC libraries were prepared as described previously^78^ and sequenced on an Illumina HiSeqX system with paired-end, 150-bp reads to ~401× sequence depth of the genome (Supplementary Fig. 2). The initial de novo assembly, raw genomic reads, and Dovetail HiC library reads were used as input data for HiRise, a software pipeline designed specifically for using proximity-ligation data to scaffold genome assemblies to chromosome-length pseudomolecules^79^. After HiRise scaffolding, the sequences were gap filled with PacBio reads with PBJelly^33^. Gaps filled with PacBio sequences were polished with Pilon (v 1.22)^80^ with Illumina paired-end data. Illumina reads were quality-trimmed with Trimmomatic^81^ and aligned to the draft contigs with bowtie2 (v 2.3.0)^82^ with default parameters. Parameters for Pilon were modified as follows: --flank 7, --K 49, and --mindepth 20. Pilon was run recursively three times, and there were minimal corrections in the third round, thus supporting accurate indel correction. A published genetic map^34^ and syntenic analyses against the *F. vesca*^37^ genomes with SynMap within CoGe^83^ were used to identify any assembly errors and haplotype variants, and to assign homoeologous chromosomes sets. Additional assembly details and results are summarized in the supplementary information.

### Tissue collection, RNA library preparation, and sequencing

Plant tissue samples (flower before anthesis, flower at anthesis, leaf collected during the day and at night, leaves treated with methyl jasmonate (30 min, 4 h, and 24 h after treatment), runner, and salt-treated and untreated roots) were collected from *Fragaria* × *ananassa* cultivar ‘Camarosa’ grown in a growth chamber and immediately flash frozen in liquid nitrogen. Leaf tissues were also collected from wild diploid species grown in a growth chamber for phylogenetic analyses (Supplementary Table 7). Total RNA was isolated with a KingFisher Pure RNA Plant Kit (Thermo Fisher) and quantified with a Qubit 3 fluorometer (Thermo Fisher). RNA libraries were prepared with the KAPA mRNA HyperPrep Kit protocol (KAPA Biosystems). All samples were submitted to the Michigan State University Research Technology Support Facility Genomics core and sequenced with paired-end, 150-bp reads on an Illumina HiSeq 4000 system.

### Transcriptome assembly and translation

Reads were cleaned with Trimmomatic v 0.32 (ref. ^81^) with adaptor trimming for TruSeq3 paired-end reads with a 1-bp mismatch, a palindrome clip threshold of 30, and a simple clip threshold of 10. Reads were then filtered on the basis of an average phred score calculated from a sliding window of 10 bp with a minimum threshold of 20 (Supplementary Dataset 4). The quality of trimmed reads was assessed afterward with FastQC^84^. Genome-guided and de novo transcriptome assemblies were generated with Trinity v 2.2.0 (ref. ^85^) for the genome annotation/expression and phylogenetic analyses, respectively. For genome annotation and expression analyses, reads were aligned to the *Fragaria* × *ananassa* cultivar ‘Camarosa’ genome with STAR v 2.5.3a^86^ with default options, except for --alignIntronMax, which was set to 10000. For genome annotation, the coordinate-sorted BAM output files from STAR were used for the genome-guided transcriptome assembly, and name-sorted SAM files were used for gene expression analysis (HTSeq in section 3). For the diploid species libraries used in the phylogenetic analyses, because transcriptome libraries were generated with a stranded method, the ‘SS_lib_type’ parameter with ‘RF’ option was used in the assembly. In addition, reads were normalized to a maximum read coverage of 100 with ‘normalize_max_read_cov’ in Trinity. The normalization option, which decreases the quantity of input reads for highly expressed genes, was used to improve assembly efficiency^87^. For homoeolog expression bias (HEB) analyses (described in the section below), counts of uniquely mapping reads were generated with HTSeq v 0.6.1 (ref. ^88^) with default options of htseq-count, except for feature type, which was set to ‘gene’ for all RNA-seq datasets of ‘Camarosa’. The fragments per kilobase per million reads mapped (FPKM) values were derived with the standard formula for FPKM = (read count/’per million’ scaling factor)/gene length in kilobases. For phylogenetic analysis, according to McKain et al.^89^, reads were aligned to the assembled transcripts with bowtie v 1.1.0 (ref. ^90^), and transcript abundance was estimated with RSEM v 1.2.29 (ref. ^91^) through the align_and_estimate_abundance.pl script packaged with Trinity. Transcripts were filtered by FPKM, an output from the aforementioned Perl script, with a minimum threshold of 1.0% of fragments per isoform mapped, as implemented in the filter_fasta_by_rsem_values.pl script. Filtered transcripts were BLASTed against the *Fragaria vesca* v 2.01 coding sequences with TBLASTX with a minimum *e* value of 1 × 10^–10^. The RefTrans package (see URLs) was used to translate assembled transcripts by filtering BLAST hits to identify the best hit with at least 75% bidirectional overlap between the transcript and *F. vesca* coding sequences. Best hits were used to guide translations with GeneWise (Wise2 v 2.2.0)^92^. The longest translations were used in downstream analyses.

### Gene annotation

The genome was annotated with the MAKER-P annotation pipeline^36^. Protein sequences (Araport11 and UniprotKB plant database), expressed sequence tags (NCBI), and ten mRNA-seq datasets (described below) and additional RNA-seq data for *Fragaria* × *ananassa* downloaded from NCBI-SRA (BioProject PRJNA394190; red ripening fruit) were used as evidence during annotation. The RNA-seq datasets were assembled into transcripts through the StringTie genome-guided approach^93^. A custom repeat library (‘Repeat annotation’ section below) and MAKER repeat library^94^ were used for genome masking. Ab initio gene prediction was performed with the gene predictors SNAP^95^ and Augustus^96^, which were previously iteratively trained for *F. vesca*^37^. During annotation, gene models with annotation edit distance <1.0 were included in the MAKER gene set and scanned for the presence of protein domains. The predicted gene models were further filtered to remove those with TE-related domains. Briefly, the protein-coding genes were searched (BLASTp, *e* = 10^–10^) against a transposase database from a previous study^36^, and if more than 50% of gene length aligned to the transposases, the gene was removed from the gene set. However, if 60% or more of the amino acid matches were due to only three individual amino acids, the alignment was considered to be caused by low complexity and was excluded. In addition, to assess whether core plant genes were annotated, the gene set was searched against the BUSCO v 2 (ref. ^35^) plant dataset (embryophyta_odb9). lncRNAs, including long intergenic noncoding RNAs, antisense overlapping transcripts, and sense overlapping transcripts, were identified with the Evolinc lncRNA-discovery pipeline (v 1.5.1)^97^. Transcripts with fewer than three reads per base pair were discarded. Putative lncRNAs with similarity (BLASTn *e* value <1 × 10^10^) to known TEs or rFAM’s catalog (v 13.0)^98^ of housekeeping RNAs were removed.

### Repeat annotation

The *Fragaria* × *ananassa* genome was searched for LTR-RTs with LTRharvest^99^ with parameters ‘-minlenltr 100 -maxlenltr 7000 -mintsd 4 -maxtsd 6 -motif TGCA -motifmis 1 -similar 85 -vic 10 -seed 20 -seqids yes’ and LTR_finder^100^ with parameters ‘-D 15000 -d 1000 -L 7000 -l 100 -p 20 -M 0.9’. The identified LTR-RT candidates were filtered with LTR_retriever^101^ with default parameters. Miniature inverted TEs (MITEs) were identified with MITE-Hunter^102^. Candidate MITEs were manually checked for TSD and TIR, which were used for superfamily classification. Those with ambiguous TSD and TIR were classified as unknowns. The *Fragaria* × *ananassa* genome was then masked with both MITE and LTR libraries through Repeatmasker^103^ (see URLs), and other repetitive elements were identified with Repeatmodeler^104^ (see URLs). The repeats were then grouped into two categories: sequences of known identity and sequences of unknown identity. The latter were then searched against the transposase database, and if they had a match, they were included in the TE library. The library was further filtered with ProtExcluder^36^ and an in-house Perl script to exclude gene fragments. The final TE library was used to annotate the *Fragaria* × *ananassa* genome with RepeatMasker^103^ with parameters ‘-q -no_is -norna -nolow -div 40’. Annotation results were summarized with the ‘famcoverage.pl’ script from the LTR-retriever package^101^.

### Organellar genome annotation

The chloroplast genome was annotated with Verdant, a web-based software suite specifically designed for plant chloroplast genomes^105^. Automated annotation of protein-coding genes, tRNAs, and rRNAs was completed with annoBTD (see URLs). Five Rosaceae plastomes in the Verdant database were selected as a reference for annotation, including the *Fragaria vesca* ‘Hawaii 4’ chloroplast genome^37^. The previously identified ORFs were BLASTed against the reference genomes with TBLASTX^106^ with an *e*-value cutoff of 0.1 and a cutoff of 50% identity between references and high-scoring segment pairs. The best reference for each ORF was used for annotation. An optimized BLASTN^106^ was used to identify and annotate tRNAs and rRNAs on the basis of reference genomes. The best-scoring references were used to annotate the RNA. Finally, the boundaries of each feature was identified on the basis of the sequence and positional information for the orthologous features from the five reference chloroplast genomes (Supplementary Fig. 5). The mitochondrial genome was annotated with the webserver for Mitofy (see URLs), a program designed to annotate the genes and tRNAs in the mitochondrial genomes of seed plants^107^. Mitofy uses NCBI-BLASTX to annotated genes on the basis of databases of 41 protein-coding genes and uses NCBI-BLASTN and tRNAscan-SE^108^ to annotate tRNAs and rRNAs on the basis of databases of 27 tRNAs and 3 rRNAs found in seed-plant mitochondrial plant genomes. The annotated plastid and mitochondrial genomes have been deposited in Dryad (see URLs).

### Synteny and comparative genomics

The ‘Camarosa’ and *F. vesca*^37^ genomes were aligned in CoGe’s SynMap program with LAST^83^. The maximum distance between two matches was set to 20 genes, and the minimum number of aligned pairs was set to ten genes. Neighboring syntenic blocks were merged with ‘Quota Align Merge’^109^, with the maximum distance between two blocks set to 40 genes. Syntenic depth was calculated with ‘Quota Align’, and the ratio of coverage depth for *F. vesca* to *F. ananassa* gene was set to 1:4. Tandemly duplicated genes were identified and filtered from CoGe outputs with a max distance of ten genes. Fractionation bias was then calculated, with the maximum query chromosomes set to 28 and the maximum target chromosomes set to seven. The analyses can be regenerated with CoGe (see URLs). The two genomes were also aligned with MUMmer v 3.2 (ref. ^110^) to identify homoeologous exchanges (Supplementary Table 10) with parameters (nucmer --maxmatch -l 80 -c 200) and visualized with dotPlotly (see URLs).

### Phylogenetic analyses

Translated transcriptomes and whole-genome protein-coding genes for *Fragaria* × *ananassa*, *F. vesca* v 2.01, *A. thaliana* TAIR10 (ref. ^111^), and *Malus domestica* v 1.0 (ref. ^112^) (Phytozome v 12)^113^ were orthogrouped with Orthofinder v 0.3 (ref. ^114^) with Diamond v 0.8.36 (ref. ^115^) for similarity searches. Orthogroups were filtered so that a minimum of five unique accessions were present. Coding sequences and amino acid translations were separated into orthogroup-specific FASTA files. Amino acid sequences were aligned with MAFFT v 7.215 (ref. ^116^) with the ‘auto’ parameter, and PAL2NAL v 14 (ref. ^117^) was used under default parameters to create a codon alignment from MAFFT-aligned amino acids. Codon alignments were filtered by removal of alignment columns with 90% or more gaps and transcripts with unaligned lengths less than 30% of the alignment length, with scripts provided with McKain et al.^89^. Orthogroup trees were reconstructed with RAxML v 8.0.6 with 500 bootstrap replicates under the GTR + gamma evolutionary model. All 108,087 protein-coding genes from the *F*. x *ananassa* ‘Camarosa’ genome were used in the initial orthogrouping. After the filtering of orthogroups with fewer than five taxa, 51,737 ‘Camarosa’ genes remained in 8,405 gene trees. A total of 19,302 unique loci identified in large syntenic blocks forming 18,839 paralogous pairs were used to assess the evolutionary history of the subgenomes. Outgroups were chosen from either *A. thaliana* or *M. domestica*, with preference given to *A. thaliana* as an outgroup. To assess the evolutionary history of octoploid strawberry’s subgenomes, a novel tree-searching algorithm was developed called ‘phylogenetic identification of subgenomes’ (PhyDS; see URLs). The only parameters needed for PhyDS are a list of taxa, if any, to ignore in the gene trees and a minimum bootstrap value to set the threshold for acceptable subtrees. In this analysis, only genes from the ‘Camarosa’ genome were ignored (that is, PhyDS did not stop when it encountered an Fxa gene other than a sister paralog) to identify each of the diploid progenitors of octoploid strawberry. Results from varying bootstrap support cutoffs are provided. These homoeologs were than mapped back to each of the assembled chromosomes and, on the basis of their relative frequencies, used to assign each chromosome to a diploid progenitor species (Supplementary Table 8).

### Gene expression analyses

HEB was assessed with the likelihood-ratio tests described in ref. ^23^, by analysis of the anther, root, and leaf transcriptome data. This test consists of a set of three nested hypotheses. The null hypothesis, *H*_0_, is that the homoeologs are expressed at equal levels after normalization for gene length and sequencing depth. The first alternative hypothesis, *H*_1_, is that one of the homoeologs is more highly expressed in all tissues, such that the difference can be explained by a single scaling factor. The second alternative hypothesis, *H*_2_, is that the homoeologs are expressed unequally and inconsistently across the three tissues. Homoeolog pairs for which *H*_0_ can be rejected for *H*_1_, but *H*_1_ cannot be rejected for *H*_2_, are therefore cases in which one of the homoeologs appears to be up- or downregulated consistently throughout the organism. For the first test, the Benjamini–Hochberg^118^ correction for multiple testing was applied. For the second test, because the question was being unable to reject a hypothesis, no correction was made. Both tests used a 1% significance level. Pairwise genomic alignments, described above, were used to identify homoeologs for each of the subgenomes, retained duplicate genes from tandem duplications, and orthologous genes to *A. thaliana*^111^, on the basis of ortholog assignments in *F. vesca*^37^. Thes complete list of *Fragaria*–*Arabidopsis* orthologs was then filtered to genes with functional data in the AraGEM *Arabidopsis* metabolic^72,119^ and STRING global protein interaction network^120^. These gene lists were used to investigate subgenome- and pathway-level-specific expression in fruit with an available transcriptome dataset in NCBI-SRA (BioProject PRJNA394190) (Supplementary Dataset 2).

### Analysis of disease-resistance-gene familie

NBS-LRR genes were detected with HMMER v 3.1 (ref. ^121^) with default settings, by searching the protein sequences of the *Fragaria* × *ananassa* genome against the raw hidden Markov model for the NB-ARC-domain family downloaded from Pfam (family ID PF00931)^122^. Only genes identified by both HMMER and BLAST were used for subsequent analysis. TIR subdomains were detected with PfamScan on default settings by searching the identified NB-ARC genes against the Pfam-A hidden Markov model. The 423 Fxa NB-ARC-domain-containing proteins were batch-searched in the NCBI Conserved Domain Database (see URLs)^123^ and Pfam database (see URLs). Results from the CD database were used to assign the gene models that contained CC, TIR, RPW8, or ‘other’ (none of the three established N-terminal domains); gene models were further mapped onto the assembled octoploid genome to assign positions (Supplementary Fig. 12). The CD results were then filtered to remove established R-gene domains (CC, TIR, RPW8, LRR, and NB-ARC), thus resulting in a list of potential integrated domains (Supplementary Dataset 1). Eight Fxa proteins with predicted Sec7/ADP-ribosylation-factor and G-nucleotide-exchange-factor domains were aligned by ClustalW and FastME 2.0 (ref. ^124^), and their illustrated domain organization is displayed in Supplementary Fig. 13. The full protein sequences of the 423 Fxa NB-ARC-domain-containing proteins were aligned with MUSCLE v 3.8.31 (ref. ^125^) under default settings. This alignment was trimmed with trimAl v 1.4.rev22 build 2015-05-21 (ref. ^126^) under default settings. An unrooted maximum-likelihood tree was constructed with RAxML v 8.2.11 (ref. ^127^) with the PROTGAMMA substitution model. The tree was visualized with the APE package v 4.1 (ref. ^128^) in R v 3.3.3 (ref. ^129^) (see URLs).

### Statistical analysis

The comparison of homoeolog-expression abundance between the dominant subgenome and the three submissive subgenomes was carried out with a likelihood-ratio test and combined with Benjamini–Hochberg correction for multiple testing with a 1% significance level. The Kolmogorov–Smirnov test was used to determine which subgenome had the lowest-overall TE densities near genes. The *χ*^2^ test, with three degrees of freedom, was used to analyze the subgenome bias of disease-resistance genes. Bootstrapping, with 500 replicates under the GTR + gamma evolutionary model, was used to assess node support in trees generated by phylogenetic analyses.

### Reporting Summary

Further information on research design is available in the Nature Research Reporting Summary linked to this article.

## Online content

Any methods, additional references, Nature Research reporting summaries, source data, statements of data availability and associated accession codes are available at 10.1038/s41588-019-0356-4.

## Supplementary information

Supplementary Text and Figures Supplementary Note, Supplementary Figures 1–16 and Supplementary Tables 1–10

Reporting Summary

Supplementary Dataset 1 Supplementary Dataset 1

Supplementary Dataset 2 Supplementary Dataset 2

Supplementary Dataset 3 Supplementary Dataset 3

Supplementary Dataset 4 Supplementary Dataset 4

## Data Availability

The genome assembly, annotation files, alignments, and phylogenetic trees are available on Dryad (see URLs). Custom software for running PhyDS phylogenetic analyses is available on GitHub (see URLs). The genome assembly and annotation files are also available on the Genome Database for Rosaceae (GDR; see URLs) and the CyVerse CoGe platform (see URLs). ‘Camarosa’ clones are available from most strawberry nurseries. The raw sequence data are available in the Sequence Read Archive under NCBI BioProject PRJNA508389 (see URLs).

## References

[1] Duchesne, A.-N. *Histoire Naturelle des Fraisiers Contenant les Vues d’Économie Réunies à la Botanique, et Suivie de Remarques Particulières sur Plusieurs Points qui ont Rapport à l’Histoire Naturelle Générale, par M. Duchesne Fils*. (Didot le Jeune, Paris, 1766).

[2] Njuguna, W., Liston, A., Cronn, R., Ashman, T.-L. & Bassil, N. Insights into phylogeny, sex function and age of *Fragaria* based on whole chloroplast genome sequencing. *Mol. Phylogenet. Evol.* **66**, 17–29 (2013). 22982444 10.1016/j.ympev.2012.08.026

[3] Tennessen, J. A., Govindarajulu, R., Ashman, T.-L. & Liston, A. Evolutionary origins and dynamics of octoploid strawberry subgenomes revealed by dense targeted capture linkage maps. *Genome Biol. Evol.* **6**, 3295–3313 (2014). 25477420 10.1093/gbe/evu261 PMC4986458

[4] McClintock, B. The significance of responses of the genome to challenge. *Science* **226**, 792–801 (1984). 15739260 10.1126/science.15739260

[5] Folta, K. M. & Gardiner, S. E. *Genetics and Genomics of Rosaceae* (Springer, New York, 2009).

[6] Staudt, G. Taxonomic studies in the genus *Fragaria* typification of *Fragaria* species known at the time of Linnaeus. *Can. J. Bot.* **40**, 869–886 (1962).

[7] Liston, A., Cronn, R. & Ashman, T.-L. *Fragaria*: a genus with deep historical roots and ripe for evolutionary and ecological insights. *Am. J. Bot.* **101**, 1686–1699 (2014). 25326614 10.3732/ajb.1400140

[8] Bringhurst, R. S. & Khan, D. A. Natural pentaploid *Fragaria chiloensis*-*F. vesca* hybrids in coastal california and their significance in polyploid *Fragaria* evolution. *Am. J. Bot.* **50**, 658–661 (1963).

[9] Milne, R. I. & Abbott, R. J. Reproductive isolation among two interfertile *Rhododendron* species: low frequency of post-F1 hybrid genotypes in alpine hybrid zones. *Mol. Ecol.* **17**, 1108–1121 (2008). 18261051 10.1111/j.1365-294X.2007.03643.x

[10] Soltis, P. S. Ancient and recent polyploidy in angiosperms. *New Phytol.* **166**, 5–8 (2005). 15760346 10.1111/j.1469-8137.2005.01379.x

[11] Freeling, M. & Thomas, B. C. Gene-balanced duplications, like tetraploidy, provide predictable drive to increase morphological complexity. *Genome Res.* **16**, 805–814 (2006). 16818725 10.1101/gr.3681406

[12] Doyle, J. J. et al. Evolutionary genetics of genome merger and doubling in plants. *Annu. Rev. Genet.* **42**, 443–461 (2008). 18983261 10.1146/annurev.genet.42.110807.091524

[13] Van de Peer, Y., Mizrachi, E. & Marchal, K. The evolutionary significance of polyploidy. *Nat. Rev. Genet.* **18**, 411–424 (2017). 28502977 10.1038/nrg.2017.26

[14] Stebbins, G. L. Jr. Types of polyploids; their classification and significance. *Adv. Genet.* **1**, 403–429 (1947). 20259289 10.1016/s0065-2660(08)60490-3

[15] Comai, L. The advantages and disadvantages of being polyploid. *Nat. Rev. Genet.* **6**, 836–846 (2005). 16304599 10.1038/nrg1711

[16] Leitch, A. R. & Leitch, I. J. Genomic plasticity and the diversity of polyploid plants. *Science* **320**, 481–483 (2008). 18436776 10.1126/science.1153585

[17] Paterson, A. H. & Wendel, J. F. Unraveling the fabric of polyploidy. *Nat. Biotechnol.* **33**, 491–493 (2015). 25965758 10.1038/nbt.3217

[18] Osborn, T. C. The contribution of polyploidy to variation in *Brassica* species. *Physiol. Plant.* **121**, 531–536 (2004).

[19] Ulrich, D. & Olbricht, K. Diversity of volatile patterns in sixteen *Fragaria vesca* L. accessions in comparison to cultivars of *Fragaria*×*ananassa*. *J. Appl. Bot. Food Qual.* **86**, 37–46 (2013).

[20] Schnable, J. C., Springer, N. M. & Freeling, M. Differentiation of the maize subgenomes by genome dominance and both ancient and ongoing gene loss. *Proc. Natl Acad. Sci. USA* **108**, 4069–4074 (2011). 21368132 10.1073/pnas.1101368108 PMC3053962

[21] Thomas, B. C., Pedersen, B. & Freeling, M. Following tetraploidy in an *Arabidopsis* ancestor, genes were removed preferentially from one homeolog leaving clusters enriched in dose-sensitive genes. *Genome Res.* **16**, 934–946 (2006). 16760422 10.1101/gr.4708406 PMC1484460

[22] Freeling, M. et al. Fractionation mutagenesis and similar consequences of mechanisms removing dispensable or less-expressed DNA in plants. *Curr. Opin. Plant. Biol.* **15**, 131–139 (2012). 22341793 10.1016/j.pbi.2012.01.015

[23] Edger, P. P. et al. Subgenome dominance in an interspecific hybrid, synthetic allopolyploid, and a 140-year-old naturally established neo-allopolyploid monkeyflower. *Plant Cell* **29**, 2150–2167 (2017). 28814644 10.1105/tpc.17.00010 PMC5635986

[24] Bottani, S., Zabet, N. R., Wendel, J. F. & Veitia, R. A. Gene expression dominance in allopolyploids: hypotheses and models. *Trends Plant Sci.* **23**, 393–402 (2018). 29433919 10.1016/j.tplants.2018.01.002

[25] Bird, K. A., VanBuren, R., Puzey, J. R. & Edger, P. P. The causes and consequences of subgenome dominance in hybrids and recent polyploids. *New Phytol.* **220**, 87–93 (2018). 29882360 10.1111/nph.15256

[26] Lysak, M. A., Cheung, K., Kitschke, M. & Bureš, P. Ancestral chromosomal blocks are triplicated in Brassiceae species with varying chromosome number and genome size. *Plant Physiol.* **145**, 402–410 (2007). 17720758 10.1104/pp.107.104380 PMC2048728

[27] Xiong, Z., Gaeta, R. T. & Pires, J. C. Homoeologous shuffling and chromosome compensation maintain genome balance in resynthesized allopolyploid *Brassica napus*. *Proc. Natl Acad. Sci. USA* **108**, 7908–7913 (2011). 21512129 10.1073/pnas.1014138108 PMC3093481

[28] Chalhoub, B. et al. Early allopolyploid evolution in the post-Neolithic *Brassica napus* oilseed genome. *Science* **345**, 950–953 (2014). 25146293 10.1126/science.1253435

[29] Edger, P. P., McKain, M. R., Bird, K. A. & VanBuren, R. Subgenome assignment in allopolyploids: challenges and future directions. *Curr. Opin. Plant Biol.* **42**, 76–80 (2018). 29649616 10.1016/j.pbi.2018.03.006

[30] Collard, B. C. Y. & Mackill, D. J. Marker-assisted selection: an approach for precision plant breeding in the twenty-first century. *Phil. Trans. R. Soc. Lond. B* **363**, 557–572 (2008). 17715053 10.1098/rstb.2007.2170 PMC2610170

[31] Hirakawa, H. et al. Dissection of the octoploid strawberry genome by deep sequencing of the genomes of *Fragaria* species. *DNA Res.* **21**, 169–181 (2014). 24282021 10.1093/dnares/dst049 PMC3989489

[32] Avni, R. et al. Wild emmer genome architecture and diversity elucidate wheat evolution and domestication. *Science* **357**, 93–97 (2017). 28684525 10.1126/science.aan0032

[33] English, A. C. et al. Mind the gap: upgrading genomes with Pacific Biosciences RS long-read sequencing technology. *PLoS One* **7**, e47768 (2012). 23185243 10.1371/journal.pone.0047768 PMC3504050

[34] Davik, J. et al. A ddRAD based linkage map of the cultivated strawberry, *Fragaria*×*ananassa*. *PLoS One* **10**, e0137746 (2015). 26398886 10.1371/journal.pone.0137746 PMC4580419

[35] Simão, F. A., Waterhouse, R. M., Ioannidis, P., Kriventseva, E. V. & Zdobnov, E. M. BUSCO: assessing genome assembly and annotation completeness with single-copy orthologs. *Bioinformatics* **31**, 3210–3212 (2015). 26059717 10.1093/bioinformatics/btv351

[36] Campbell, M. S. et al. MAKER-P: a tool kit for the rapid creation, management, and quality control of plant genome annotations. *Plant Physiol.* **164**, 513–524 (2014). 24306534 10.1104/pp.113.230144 PMC3912085

[37] Edger, P. P. et al. Single-molecule sequencing and optical mapping yields an improved genome of woodland strawberry (*Fragaria vesca*) with chromosome-scale contiguity. *Gigascience* **7**, 1–7 (2018). 29253147 10.1093/gigascience/gix124 PMC5801600

[38] Potter, D., Luby, J. J. & Harrison, R. E. Phylogenetic relationships among species of *Fragaria* (Rosaceae) inferred from non-coding nuclear and chloroplast DNA sequences. *Syst. Bot.* **25**, 337–348 (2000).

[39] Yang, Y. & Davis, T. M. A new perspective on polyploid *Fragaria* (strawberry) genome composition based on large-scale, multi-locus phylogenetic analysis. *Genome Biol. Evol.* **9**, 3433–3448 (2017). 29045639 10.1093/gbe/evx214 PMC5751083

[40] Fedorova, N. J. Crossability and phylogenetic relations in the main European species of *Fragaria*. *Compil. Natl Acad. Sci. USSR.* **52**, 545–547 (1946).

[41] Lundberg, M. *Systematics and Polyploid Evolution in Potentilleae (Rosaceae)*. PhD thesis, Stockholm University (2011).

[42] Johnson, A. L., Govindarajulu, R. & Ashman, T.-L. Bioclimatic evaluation of geographical range in *Fragaria* (Rosaceae): consequences of variation in breeding system, ploidy and species age. *Bot. J. Linn. Soc.* **176**, 99–114 (2014).

[43] Sankoff, D., Zheng, C. & Wang, B. A model for biased fractionation after whole genome duplication. *BMC Genomics* **13** (Suppl. 1), S8 (2012). 10.1186/1471-2164-13-S1-S8 PMC3471344 22369177

[44] Wang, X. et al. The genome of the mesopolyploid crop species *Brassica rapa*. *Nat. Genet.* **43**, 1035–1039 (2011). 21873998 10.1038/ng.919

[45] Eckardt, N. A. Genome dominance and interaction at the gene expression level in allohexaploid wheat. *Plant Cell* **26**, 1834 (2014). 24838977 10.1105/tpc.114.127183 PMC4079349

[46] Parkin, I. A. P. et al. Transcriptome and methylome profiling reveals relics of genome dominance in the mesopolyploid *Brassica oleracea*. *Genome. Biol.* **15**, R77 (2014). 24916971 10.1186/gb-2014-15-6-r77 PMC4097860

[47] Cheng, F. et al. Epigenetic regulation of subgenome dominance following whole genome triplication in *Brassica rapa*. *New Phytol.* **211**, 288–299 (2016). 26871271 10.1111/nph.13884

[48] Renny-Byfield, S., Rodgers-Melnick, E. & Ross-Ibarra, J. Gene fractionation and function in the ancient subgenomes of maize. *Mol. Biol. Evol.* **34**, 1825–1832 (2017). 28430989 10.1093/molbev/msx121

[49] Woodhouse, M. R. et al. Origin, inheritance, and gene regulatory consequences of genome dominance in polyploids. *Proc. Natl Acad. Sci. USA* **111**, 5283–5288 (2014). 24706847 10.1073/pnas.1402475111 PMC3986174

[50] Garsmeur, O. et al. Two evolutionarily distinct classes of paleopolyploidy. *Mol. Biol. Evol.* **31**, 448–454 (2014). 24296661 10.1093/molbev/mst230

[51] Zhao, M., Zhang, B., Lisch, D. & Ma, J. Patterns and consequences of subgenome differentiation provide insights into the nature of paleopolyploidy in plants. *Plant Cell* **29**, 2974–2994 (2017). 29180596 10.1105/tpc.17.00595 PMC5757279

[52] Wendel, J. F., Lisch, D., Hu, G. & Mason, A. S. The long and short of doubling down: polyploidy, epigenetics, and the temporal dynamics of genome fractionation. *Curr. Opin. Genet. Dev.* **49**, 1–7 (2018). 29438956 10.1016/j.gde.2018.01.004

[53] Douglas, G. M. et al. Hybrid origins and the earliest stages of diploidization in the highly successful recent polyploid *Capsella bursa-pastoris*. *Proc. Natl Acad. Sci. USA* **112**, 2806–2811 (2015). 25691747 10.1073/pnas.1412277112 PMC4352811

[54] Sun, H. et al. Karyotype stability and unbiased fractionation in the paleo-allotetraploid *Cucurbita* genomes. *Mol. Plant* **10**, 1293–1306 (2017). 28917590 10.1016/j.molp.2017.09.003

[55] Osborn, T. C. et al. Understanding mechanisms of novel gene expression in polyploids. *Trends Genet.* **19**, 141–147 (2003). 12615008 10.1016/s0168-9525(03)00015-5

[56] Hollister, J. D. & Gaut, B. S. Epigenetic silencing of transposable elements: a trade-off between reduced transposition and deleterious effects on neighboring gene expression. *Genome Res.* **19**, 1419–1428 (2009). 19478138 10.1101/gr.091678.109 PMC2720190

[57] Rizzon, C., Ponger, L. & Gaut, B. S. Striking similarities in the genomic distribution of tandemly arrayed genes in *Arabidopsis* and rice. *PLoS Comput. Biol.* **2**, e115 (2006). 16948529 10.1371/journal.pcbi.0020115 PMC1557586

[58] Hanada, K., Zou, C., Lehti-Shiu, M. D., Shinozaki, K. & Shiu, S.-H. Importance of lineage-specific expansion of plant tandem duplicates in the adaptive response to environmental stimuli. *Plant Physiol.* **148**, 993–1003 (2008). 18715958 10.1104/pp.108.122457 PMC2556807

[59] Qian, L.-H. et al. Distinct patterns of gene gain and loss: diverse evolutionary modes of NBS-encoding genes in three solanaceae crop species. *G3 (Bethesda)* **7**, 1577–1585 (2017). 28364035 10.1534/g3.117.040485 PMC5427506

[60] Meyers, B. C., Kozik, A., Griego, A., Kuang, H. & Michelmore, R. W. Genome-wide analysis of NBS-LRR-encoding genes in *Arabidopsis*. *Plant Cell* **15**, 809–834 (2003). 12671079 10.1105/tpc.009308 PMC152331

[61] Dangl, J. L., Horvath, D. M. & Staskawicz, B. J. Pivoting the plant immune system from dissection to deployment. *Science* **341**, 746–751 (2013). 23950531 10.1126/science.1236011 PMC3869199

[62] Kroj, T., Chanclud, E., Michel-Romiti, C., Grand, X. & Morel, J.-B. Integration of decoy domains derived from protein targets of pathogen effectors into plant immune receptors is widespread. *New Phytol.* **210**, 618–626 (2016). 26848538 10.1111/nph.13869 PMC5067614

[63] Sarris, P. F., Cevik, V., Dagdas, G., Jones, J. D. G. & Krasileva, K. V. Comparative analysis of plant immune receptor architectures uncovers host proteins likely targeted by pathogens. *BMC Biol.* **14**, 8 (2016). 26891798 10.1186/s12915-016-0228-7 PMC4759884

[64] Roach, J. A. et al. FaRXf1: a locus conferring resistance to angular leaf spot caused by *Xanthomonas fragariae* in octoploid strawberry. *Theor. Appl. Genet.* **129**, 1191–1201 (2016). 26910360 10.1007/s00122-016-2695-1

[65] Mangandi, J. et al. Pedigree-based analysis in a multiparental population of octoploid strawberry reveals QTL alleles conferring resistance to P*hytophthora cactorum*. *G3 (Bethesda)* **7**, 1707–1719 (2017). 28592652 10.1534/g3.117.042119 PMC5473751

[66] Pincot, D. D. A. et al. Genome-wide association mapping uncovers Fw1, a dominant gene conferring resistance to Fusarium wilt in strawberry. *G3 (Bethesda)* **8**, 1817–1828 (2018). 29602808 10.1534/g3.118.200129 PMC5940171

[67] Guo, H. et al. Extensive and biased intergenomic nonreciprocal DNA exchanges shaped a nascent polyploid genome, *Gossypium* (cotton). *Genetics* **197**, 1153–1163 (2014). 24907262 10.1534/genetics.114.166124 PMC4125390

[68] Gaeta, R. T. & Chris Pires, J. Homoeologous recombination in allopolyploids: the polyploid ratchet. *New Phytol.* **186**, 18–28 (2010). 20002315 10.1111/j.1469-8137.2009.03089.x

[69] Zhang, T. et al. Sequencing of allotetraploid cotton (*Gossypium hirsutum* L. acc. TM-1) provides a resource for fiber improvement. *Nat. Biotechnol.* **33**, 531–537 (2015). 25893781 10.1038/nbt.3207

[70] He, Z. et al. Extensive homoeologous genome exchanges in allopolyploid crops revealed by mRNAseq-based visualization. *Plant. Biotechnol. J.* **15**, 594–604 (2017). 27808473 10.1111/pbi.12657 PMC5399007

[71] Birchler, J. A., Bhadra, U., Bhadra, M. P. & Auger, D. L. Dosage-dependent gene regulation in multicellular eukaryotes: implications for dosage compensation, aneuploid syndromes, and quantitative traits. *Dev. Biol.* **234**, 275–288 (2001). 11396999 10.1006/dbio.2001.0262

[72] Bekaert, M., Edger, P. P., Pires, J. C. & Conant, G. C. Two-phase resolution of polyploidy in the *Arabidopsis* metabolic network gives rise to relative and absolute dosage constraints. *Plant Cell* **23**, 1719–1728 (2011). 21540436 10.1105/tpc.110.081281 PMC3123947

[73] Laricchia, K. M., Zdraljevic, S., Cook, D. E. & Andersen, E. C. Natural variation in the distribution and abundance of transposable elements across the *Caenorhabditis elegans* Species. *Mol. Biol. Evol.* **34**, 2187–2202 (2017). 28486636 10.1093/molbev/msx155 PMC5850821

[74] Yang, J. et al. The genome sequence of allopolyploid *Brassica juncea* and analysis of differential homoeolog gene expression influencing selection. *Nat. Genet.* **48**, 1225–1232 (2016). 27595476 10.1038/ng.3657

[75] Zhang, H.-B., Zhao, X., Ding, X., Paterson, A. H. & Wing, R. A. Preparation of megabase-size DNA from plant nuclei. *Plant J.* **7**, 175–184 (1995).

[76] VanBuren, R. et al. Single-molecule sequencing of the desiccation-tolerant grass *Oropetium thomaeum*. *Nature* **527**, 508–511 (2015). 26560029 10.1038/nature15714

[77] Luo, M.-C. et al. Genome sequence of the progenitor of the wheat D genome *Aegilops tauschii*. *Nature* **551**, 498–502 (2017). 29143815 10.1038/nature24486 PMC7416625

[78] Lieberman-Aiden, E. et al. Comprehensive mapping of long-range interactions reveals folding principles of the human genome. *Science* **326**, 289–293 (2009). 19815776 10.1126/science.1181369 PMC2858594

[79] Putnam, N. H. et al. Chromosome-scale shotgun assembly using an in vitro method for long-range linkage. *Genome Res.* **26**, 342–350 (2016). 26848124 10.1101/gr.193474.115 PMC4772016

[80] Walker, B. J. et al. Pilon: an integrated tool for comprehensive microbial variant detection and genome assembly improvement. *PLoS One* **9**, e112963 (2014). 25409509 10.1371/journal.pone.0112963 PMC4237348

[81] Bolger, A. M., Lohse, M. & Usadel, B. Trimmomatic: a flexible trimmer for Illumina sequence data. *Bioinformatics* **30**, 2114–2120 (2014). 24695404 10.1093/bioinformatics/btu170 PMC4103590

[82] Langmead, B. & Salzberg, S. L. Fast gapped-read alignment with Bowtie 2. *Nat. Methods* **9**, 357–359 (2012). 22388286 10.1038/nmeth.1923 PMC3322381

[83] Lyons, E., Pedersen, B., Kane, J. & Freeling, M. The value of nonmodel genomes and an example using SynMap within CoGe to dissect the hexaploidy that predates the Rosids. *Trop. Plant Biol.* **1**, 181–190 (2008).

[84] Leggett, R. M., Ramirez-Gonzalez, R. H., Clavijo, B. J., Waite, D. & Davey, R. P. Sequencing quality assessment tools to enable data-driven informatics for high throughput genomics. *Front. Genet.* **4**, 288 (2013). 24381581 10.3389/fgene.2013.00288 PMC3865868

[85] Grabherr, M. G. et al. Full-length transcriptome assembly from RNA-Seq data without a reference genome. *Nat. Biotechnol.* **29**, 644–652 (2011). 21572440 10.1038/nbt.1883 PMC3571712

[86] Dobin, A. & Gingeras, T. R. Mapping RNA-seq reads with STAR. *Curr. Protoc. Bioinformatics.* **51**, 11.14.1–11.14.19 (2015). 10.1002/0471250953.bi1114s51 PMC4631051 26334920

[87] Haas, B. J. et al. De novo transcript sequence reconstruction from RNA-seq using the Trinity platform for reference generation and analysis. *Nat. Protoc.* **8**, 1494–1512 (2013). 23845962 10.1038/nprot.2013.084 PMC3875132

[88] Anders, S., Pyl, P. T. & Huber, W. HTSeq: a Python framework to work with high-throughput sequencing data. *Bioinformatics* **31**, 166–169 (2015). 25260700 10.1093/bioinformatics/btu638 PMC4287950

[89] McKain, M. R. et al. A phylogenomic assessment of ancient polyploidy and genome evolution across the Poales. *Genome Biol. Evol.* **8**, 1150–1164 (2016). 26988252 10.1093/gbe/evw060 PMC4860692

[90] Langmead, B., Trapnell, C., Pop, M. & Salzberg, S. L. Ultrafast and memory-efficient alignment of short DNA sequences to the human genome. *Genome. Biol.* **10**, R25 (2009). 19261174 10.1186/gb-2009-10-3-r25 PMC2690996

[91] Li, B. & Dewey, C. N. RSEM: accurate transcript quantification from RNA-Seq data with or without a reference genome. *BMC Bioinformatics* **12**, 323 (2011). 21816040 10.1186/1471-2105-12-323 PMC3163565

[92] Birney, E., Clamp, M. & Durbin, R. GeneWise and Genomewise. *Genome Res.* **14**, 988–995 (2004). 15123596 10.1101/gr.1865504 PMC479130

[93] Pertea, M. et al. StringTie enables improved reconstruction of a transcriptome from RNA-seq reads. *Nat. Biotechnol.* **33**, 290–295 (2015). 25690850 10.1038/nbt.3122 PMC4643835

[94] Cantarel, B. L. et al. MAKER: an easy-to-use annotation pipeline designed for emerging model organism genomes. *Genome Res.* **18**, 188–196 (2008). 18025269 10.1101/gr.6743907 PMC2134774

[95] Korf, I. Gene finding in novel genomes. *BMC Bioinformatics* **5**, 59 (2004). 15144565 10.1186/1471-2105-5-59 PMC421630

[96] Stanke, M. & Waack, S. Gene prediction with a hidden Markov model and a new intron submodel. *Bioinformatics* **19** (Suppl. 2), ii215–ii225 (2003). 10.1093/bioinformatics/btg1080 14534192

[97] Nelson, A. D. L. et al. Evolinc: a tool for the identification and evolutionary comparison of long intergenic non-coding RNAs. *Front. Genet.* **8**, 52 (2017). 28536600 10.3389/fgene.2017.00052 PMC5422434

[98] Kalvari, I. et al. Rfam 13.0: shifting to a genome-centric resource for non-coding RNA families. *Nucleic Acids Res.* **46**, D335–D342 (2018). 29112718 10.1093/nar/gkx1038 PMC5753348

[99] Ellinghaus, D., Kurtz, S. & Willhoeft, U. LTRharvest, an efficient and flexible software for de novo detection of LTR retrotransposons. *BMC Bioinformatics* **9**, 18 (2008). 18194517 10.1186/1471-2105-9-18 PMC2253517

[100] Xu, Z. & Wang, H. LTR_FINDER: an efficient tool for the prediction of full-length LTR retrotransposons. *Nucleic Acids Res.* **35**, W265–W268 (2007). 17485477 10.1093/nar/gkm286 PMC1933203

[101] Ou, S. & Jiang, N. LTR_retriever: a highly accurate and sensitive program for identification of long terminal repeat retrotransposons. *Plant Physiol.* **176**, 1410–1422 (2018). 29233850 10.1104/pp.17.01310 PMC5813529

[102] Han, Y. & Wessler, S. R. MITE-Hunter: a program for discovering miniature inverted-repeat transposable elements from genomic sequences. *Nucleic Acids Res.* **38**, e199 (2010). 20880995 10.1093/nar/gkq862 PMC3001096

[103] Tarailo-Graovac, M. & Chen, N. Using RepeatMasker to identify repetitive elements in genomic sequences. *Curr. Protoc. Bioinformatics.* **25**, 4.10 (2009). 10.1002/0471250953.bi0410s25 19274634

[104] Bao, L. & Liu, Z. in *Bzioinformatics in Aquaculture* (ed. Liu, Z. J.) **8**, 86–97 (Wiley, Hoboken, NJ, USA, 2017).

[105] McKain, M. R., Hartsock, R. H., Wohl, M. M. & Kellogg, E. A. Verdant: automated annotation, alignment and phylogenetic analysis of whole chloroplast genomes. *Bioinformatics* **33**, 130–132 (2017). 27634949 10.1093/bioinformatics/btw583 PMC5408774

[106] Camacho, C. et al. BLAST+: architecture and applications. *BMC Bioinformatics* **10**, 421 (2009). 20003500 10.1186/1471-2105-10-421 PMC2803857

[107] Alverson, A. J. et al. Insights into the evolution of mitochondrial genome size from complete sequences of *Citrullus lanatus* and *Cucurbita pepo* (Cucurbitaceae). *Mol. Biol. Evol.* **27**, 1436–1448 (2010). 20118192 10.1093/molbev/msq029 PMC2877997

[108] Lowe, T. M. & Eddy, S. R. tRNAscan-SE: a program for improved detection of transfer RNA genes in genomic sequence. *Nucleic Acids Res.* **25**, 955–964 (1997). 9023104 10.1093/nar/25.5.955 PMC146525

[109] Tang, H. et al. Screening synteny blocks in pairwise genome comparisons through integer programming. *BMC Bioinformatics* **12**, 102 (2011). 21501495 10.1186/1471-2105-12-102 PMC3088904

[110] Kurtz, S. et al. Versatile and open software for comparing large genomes. *Genome. Biol.* **5**, R12 (2004). 14759262 10.1186/gb-2004-5-2-r12 PMC395750

[111] Lamesch, P. et al. The *Arabidopsis* Information Resource (TAIR): improved gene annotation and new tools. *Nucleic Acids Res.* **40**, D1202–D1210 (2012). 22140109 10.1093/nar/gkr1090 PMC3245047

[112] Velasco, R. et al. The genome of the domesticated apple (*Malus* × *domestica* Borkh.). *Nat. Genet.* **42**, 833–839 (2010). 20802477 10.1038/ng.654

[113] Goodstein, D. M. et al. Phytozome: a comparative platform for green plant genomics. *Nucleic Acids Res.* **40**, D1178–D1186 (2012). 22110026 10.1093/nar/gkr944 PMC3245001

[114] Emms, D. M. & Kelly, S. OrthoFinder: solving fundamental biases in whole genome comparisons dramatically improves orthogroup inference accuracy. *Genome. Biol.* **16**, 157 (2015). 26243257 10.1186/s13059-015-0721-2 PMC4531804

[115] Buchfink, B., Xie, C. & Huson, D. H. Fast and sensitive protein alignment using DIAMOND. *Nat. Methods* **12**, 59–60 (2015). 25402007 10.1038/nmeth.3176

[116] Katoh, K. & Standley, D. M. MAFFT multiple sequence alignment software version 7: improvements in performance and usability. *Mol. Biol. Evol.* **30**, 772–780 (2013). 23329690 10.1093/molbev/mst010 PMC3603318

[117] Suyama, M., Torrents, D. & Bork, P. PAL2NAL: robust conversion of protein sequence alignments into the corresponding codon alignments. *Nucleic Acids Res.* **34**, W609–W612 (2006). 16845082 10.1093/nar/gkl315 PMC1538804

[118] Benjamini, Y. & Hochberg, Y. Controlling the false discovery rate: a practical and powerful approach to multiple testing. *J. R. Stat. Soc. Series B Stat. Methodol.* **57**, 289–300 (1995).

[119] de Oliveira Dal’Molin, C. G., Quek, L.-E., Palfreyman, R. W., Brumbley, S. M. & Nielsen, L. K. AraGEM, a genome-scale reconstruction of the primary metabolic network in. *Arabidopsis. Plant Physiol.* **152**, 579–589 (2010). 20044452 10.1104/pp.109.148817 PMC2815881

[120] Szklarczyk, D. et al. The STRING database in 2017: quality-controlled protein-protein association networks, made broadly accessible. *Nucleic Acids Res.* **45**, D362–D368 (2017). 27924014 10.1093/nar/gkw937 PMC5210637

[121] Eddy, S. R. Accelerated profile HMM searches. *PLoS Comput. Biol.* **7**, e1002195 (2011). 22039361 10.1371/journal.pcbi.1002195 PMC3197634

[122] Finn, R. D. et al. The Pfam protein families database: towards a more sustainable future. *Nucleic Acids Res.* **44** (D1), D279–D285 (2016). 26673716 10.1093/nar/gkv1344 PMC4702930

[123] Marchler-Bauer, A. et al. CDD: NCBI’s conserved domain database. *Nucleic Acids Res.* **43**, D222–D226 (2015). 25414356 10.1093/nar/gku1221 PMC4383992

[124] Lefort, V., Desper, R. & Gascuel, O. FastME 2.0: a comprehensive, accurate, and fast distance-based phylogeny inference program. *Mol. Biol. Evol.* **32**, 2798–2800 (2015). 26130081 10.1093/molbev/msv150 PMC4576710

[125] Edgar, R. C. MUSCLE: multiple sequence alignment with high accuracy and high throughput. *Nucleic Acids Res.* **32**, 1792–1797 (2004). 15034147 10.1093/nar/gkh340 PMC390337

[126] Capella-Gutiérrez, S., Silla-Martínez, J. M. & Gabaldón, T. trimAl: a tool for automated alignment trimming in large-scale phylogenetic analyses. *Bioinformatics* **25**, 1972–1973 (2009). 19505945 10.1093/bioinformatics/btp348 PMC2712344

[127] Stamatakis, A. RAxML version 8: a tool for phylogenetic analysis and post-analysis of large phylogenies. *Bioinformatics* **30**, 1312–1313 (2014). 24451623 10.1093/bioinformatics/btu033 PMC3998144

[128] Paradis, E., Claude, J. & Strimmer, K. APE: analyses of phylogenetics and evolution in R language. *Bioinformatics* 20, (289–290 (2004). 10.1093/bioinformatics/btg412 14734327

[129] Ihaka, R. & Gentleman, R. R. A language for data analysis and graphics. *J. Comput. Graph. Stat.* **5**, 299–314 (1996).

