# Supplementary material for: Origin and evolution of the octoploid strawberry genome
Source: Nat Genet. 2019 Feb 25;51(3):541–7. doi: 10.1038/s41588-019-0356-4 (PMC6882729; doi:10.1038/s41588-019-0356-4)
Supplement: Supplementary file 1 — Supplementary Note, Supplementary Figures 1–16 and Supplementary Tables 1–10 [file 41588_2019_356_MOESM1_ESM.pdf]

nature genetics

SUPPLEMENTARY INFORMATION
https://doi.org/10.1038/s41588-019-0356-4

In the format provided by the authors and unedited.

Origin and evolution of the octoploid strawberry genome

Patrick P. Edger[1,2*], Thomas J. Poorten[3], Robert VanBuren[1,4], Michael A. Hardigan[3], Marivi Colle[1], Michael R. McKain[5], Ronald D. Smith[6], Scott J. Teresi[6], Andrew D. L. Nelson[7], Ching Man Wai[1], Elizabeth I. Alger[1], Kevin A. Bird[1,2], Alan E. Yocca[1], Nathan Pumplin[3], Shujun Ou[1,2], Gil Ben-Zvi[8], Avital Brodt[8], Kobi Baruch[8], Thomas Swale[9], Lily Shiue[9], Charlotte B. Acharya[3], Glenn S. Cole[3], Jeffrey P. Mower[10], Kevin L. Childs[11,12], Ning Jiang[1,2], Eric Lyons[7], Michael Freeling[13], Joshua R. Puzey[6] and Steven J. Knapp[3*]

[1]Department of Horticulture, Michigan State University, East Lansing, MI, USA. [2]Ecology, Evolutionary Biology and Behavior, Michigan State University, East Lansing, MI, USA. [3]Department of Plant Sciences, University of California–Davis, Davis, California, USA. [4]Plant Resilience Institute, Michigan State University, East Lansing, MI, USA. [5]Department of Biological Sciences, University of Alabama, Tuscaloosa, AL, USA. [6]Department of Biology, College of William and Mary, Williamsburg, VA, USA. [7]School of Plant Sciences, University of Arizona, Tucson, AZ, USA. [8]NRGene, Ness Ziona, Israel. [9]Dovetail Genomics, Santa Cruz, CA, USA. [10]Center for Plant Science Innovation, University of Nebraska, Lincoln, NE, USA. [11]Department of Plant Biology, Michigan State University, East Lansing, MI, USA. [12]Center for Genomics Enabled Plant Science, Michigan State University, East Lansing, MI, USA. [13]Department of Plant and Microbial Biology, University of California, Berkeley, Berkeley, CA, USA. *e-mail: edgerpat@msu.edu; sjknapp@ucdavis.edu

Supplementary Information

1. Genome Assembly

1) Reads pre-processing. PCR duplicates, illumina adaptor AGATCGGAAGAGC and Nextera linkers (for MP libraries) were removed. The PE 470bp 2×265bp libraries (Supplemental Table 1) overlapping reads were merged with minimal required overlap of 10bp to create the stitched reads.
2) Error correction. Following pre-processing, merged PE reads were scanned to detect and filter reads with putative sequencing error (contain a sub-sequence that does not reappear in other reads).
3) Contigs assembly. The first step of the assembly consists of building a *de bruijn* graph (kmer=127 bp) of contigs from the all PE & MP reads (Supplemental Table 1). Next, PE reads were used to find reliable paths in the graph between contigs for repeat resolving and contigs extension. 10X barcoded reads were mapped to contigs ensure that adjacent contigs were connected only in case there is an evidence that those contigs originate from a single stretch of genomic sequence (reads from the same two or more barcodes were mapped to both contigs).
4) Scaffolds assembly. Later, contigs were linked into scaffolds with PE and MP information, estimating gaps between the contigs according to the distance of PE and MP links. In addition, 10X data was used to validate and support correct phasing during scaffolding.
5) Fill Gaps. A final fill gap step used PE and MP links and *de bruijn* graph information to detect a unique path connecting the gap edges.
6) Scaffolds elongation and refinement. 10X barcoded reads were mapped to the assembled scaffolds and clusters of reads with the same barcode mapped to adjacent contigs in the scaffolds were identified to be part of a single long molecule. Next, each scaffold was scanned with a 20kb length window to ensure that the number of distinct clusters that cover the entire window (indicating a support for this 20kb connection by several long molecules) was statistically significant with respect to the number of clusters that span the left and the right edge of the window. In case where a potential scaffold assembly error was detected the scaffold was broken at the two edges of the suspicious 20kb window. Finally, the barcodes that were mapped to the scaffold edges were compared (first and last 20kb sequences) to generate a scaffolds graph with a link connecting two scaffolds with more than two common barcodes. Linear scaffolds paths in the scaffolds graph were composed into the final scaffolds output of the assembly (Supplementary Table 2).
7) Genetic map. A RADseq based genetic map [1] was used to correct misassemblies, identified 408.16Mb of haplotype variants, and extracted four homoeologs representing each subgenome. Comparisons to the genetic map was fully supported by comparative genomics with the diploid F. vesca genome [2] (Supplemental Figure 1). Supplemental Figure 1 shows syntenic scaffolds from chromosomes 3 of 'Camarosa'. Colors depict the four diploid progenitor species. The box below is highlighting a region along chromosome 3 where all eight haplotypes were phased. The largest fragments from each subgenome was selected for subsequent scaffolding steps, while the smaller fragment was added to the 'haplotype variant' file and are available along with the remainder of the genome.
8) HiC Scaffolding. The four representative homoeologous scaffolds, raw reads, and Dovetail HiC library reads were used as input data (Supplementary Figure 2) for HiRise, a software pipeline designed specifically for using proximity ligation data to scaffold genome assemblies to chromosome-length pseudomolecules (Supplemental Figure 3). A total of 371 million 150bp paired-end reads were sequenced; equating to ~401x sequence depth across the genome.

9) Gap Filling. After HiRise scaffolding, sequences were gap filled with PacBio reads (Supplementary Table 3) using PBJelly [3], which were first error corrected using Pilon [4]. This resulted in ~7.5Mb of new sequence and bringing the total assembly size to 805,488,706bp across 28 chromosomes (Figure 1).

2. Phylogenomics

The goal of this phylogenetic analyses was to identify each of the diploid progenitor species for each of the subgenomes in octoploid strawberry (Supplemental Table 7). Thus, all twelve described diploid species, plus four *F. vesca* subspecies, collected from around the world were sampled in this study. We included multiple accessions for each species, if available, and all germplasm is available from the National Clonal Germplasm Repository (https://www.ars.usda.gov/pacific-west-area/corvallis-or/national-clonal-germplasm-repository/) located in Corvallis, OR. Polyploid intermediates were not included in this study. A total of 51,737 protein coding genes from 'Camarosa' were assessed for evolutionary history in 8,405 gene trees. Using Phylogenetic iDentification of Subgenomes (https://github.com/mrmckain/PhyDS), we identified how many of each taxon and accession were found in a clade with a Camarosa gene using various BSV cutoff values -- see Supplemental Figure 5. We also considered situations where there were only Camarosa and one other taxon in the clade to see if these data sets differed. The same trend was present with *Fragaria vesca* being the most prevalent with *F. vesca* var. *bracteata* having most instances of relatedness of all *F. vesca* subspecies (1757 subtrees; BSV50: 1135 subtrees; BSV80: 515 subtrees). *F. innumae* is the second most prevalent taxon (971 subtrees; BSV50: 718 subtrees; BSV80: 336 subtrees). *F. viridis* is the third most prevalent taxon (700 subtrees; BSV50: 459 subtrees; BSV80: 208 subtrees). The fourth most prevalent taxon is *F. nipponica* (611 subtrees; BSV50: 390 subtrees, BSV80: 159 subtrees). The hybridization sequence of the four progenitor species towards the formation of the octoploid is depicted in Supplemental Figure 7. Related diploid species were also identified (Supplemental Figure 6) for reasons outlined in Supplemental Figure 8. However, introgression via interspecific hybridization, as well as incomplete lineage sorting (ILS), are also likely sources of incongruence among some trees given that these are common evolutionary processes across higher eukaryotes including plants. However, given the relative abundance of the four identified diploid progenitors and identification of immediate sister species (e.g. *F. vesca ssp. vesca* & ssp. *californica*), majority of trees do not support an evolutionary history of ILS and/or interspecific hybridization events.

Chromosome assignments for *F. vesca* and *F. iinumae* subgenomes are congruent with a previous study using a genetic mapping approach [5]. These results support our phylogenetic approach for assigning chromosomes to each of these subgenomes. However, future studies are needed to confirm the chromosome assignments for *F. viridis* and *F. nipponica*. Reference genomes for these two diploid species are ideally needed to confirm chromosome assignments and regions of homoeologous exchanges (Supplemental Figure 16). Importantly, downstream analyses of subgenome dominance presented in the manuscript are not impacted by the chromosome assignments for the *F. viridis* and *F. nipponica* subgenomes. We largely only compare the dominant *F. vesca* subgenome to all of the other three subgenomes combined, including comparisons of gene expression, gene content, TE content, and tandem gene duplicates (see below Section 5). The three submissive subgenomes are treated individually for only the analysis of homoeologous exchanges with nearly identical results for *F. viridis* (9.8x bias) and *F.*

nipponica (10.4x bias). Any chromosome mis-assignments among these, the two most submissive subgenomes, would have no impact on the subgenome dominance patterns observed in octoploid strawberry.

Furthermore, each subgenome has undergone a history of gene loss and homoeologous exchanges since the hybridization and polyploid events (see below section 5). The two most submissive subgenomes, *F. viridis* and *F. nipponica*, have undergone the most changes since merging into the nucleus with the other two subgenomes. These chromosomes are now remnant fragments of the original chromosomes mixed with homoeologous regions derived from the other progenitor species. Thus, it's possible that some chromosomes may have been mis-assigned or possibility due to their fragmented nature may not be able to be perfectly assigned to one of these two diploid progenitor species. Chromosome assignments will be confirmed when reference genomes become available for the two extant relatives of these progenitor species.

All of the data, including the raw data and phylogenetic trees, and scripts associated with these phylogenetic analyses are available on the NCBI database under BioProject PRJNA508389, Dryad Digitial Respository (see URLs), and GitHub (see URLs).

Supplementary Figures

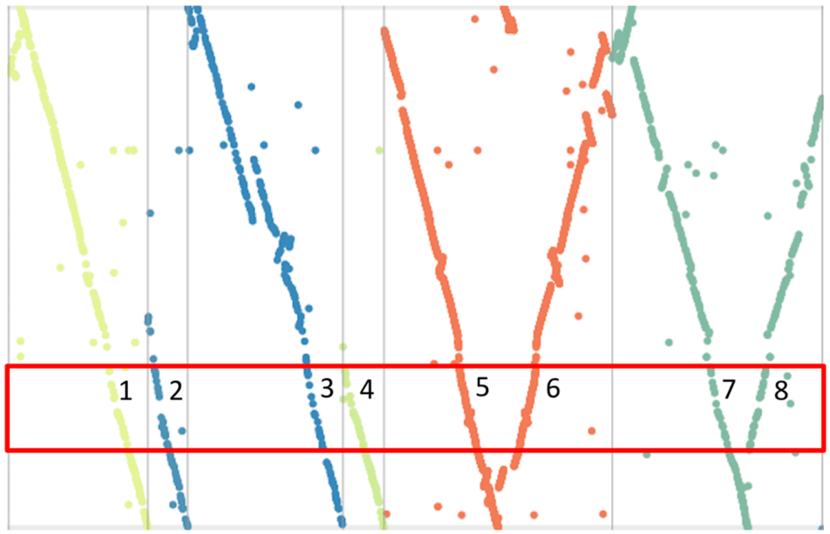

Supplemental Figure 1: Syntenic comparison of subgenomes and haplotypes of Chromosome 3. Each color (yellow, blue, red and green) is unique to each of the homoeologous chromosomes compared to *F. vesca* genome [2].

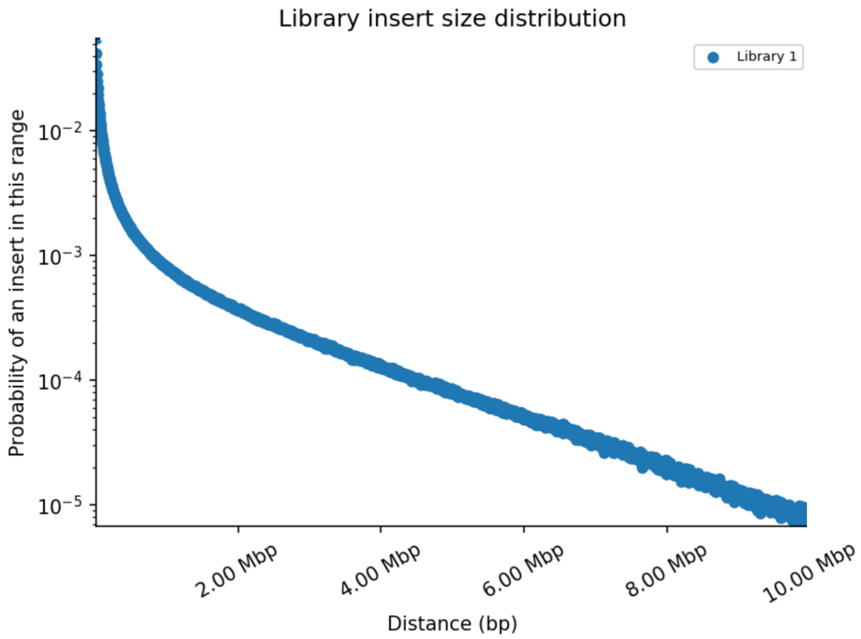

Supplementary Figure 2: Distribution of insert sizes in the Dovetail library. The distance between the forward and reverse reads is given on the X-axis in basepairs, and the probability of observing a read pair with a given insert size is shown on the Y- axis.

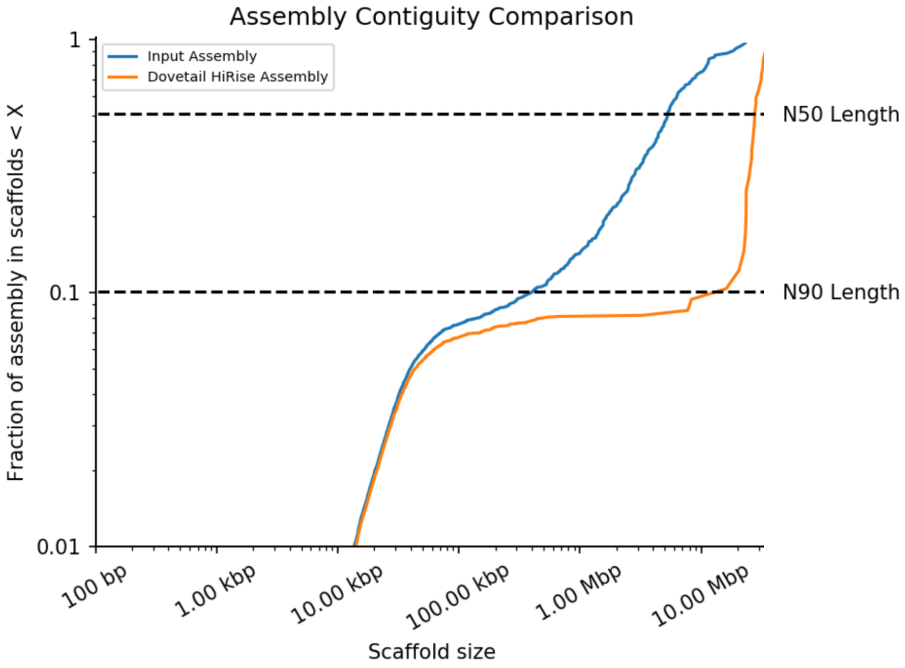

Supplemental Figure 3: A comparison of the contiguity of the input assembly (shown in blue) and the final HiRise scaffolds (shown in orange). Each curve shows the fraction of the total length of the assembly present in scaffolds of a given length or smaller. The fraction of the assembly is indicated on the Y-axis and the scaffold length in basepairs is given on the X-axis. The two dashed lines mark the N50 and N90 lengths of each assembly. Scaffolds less than 1 kb are excluded.

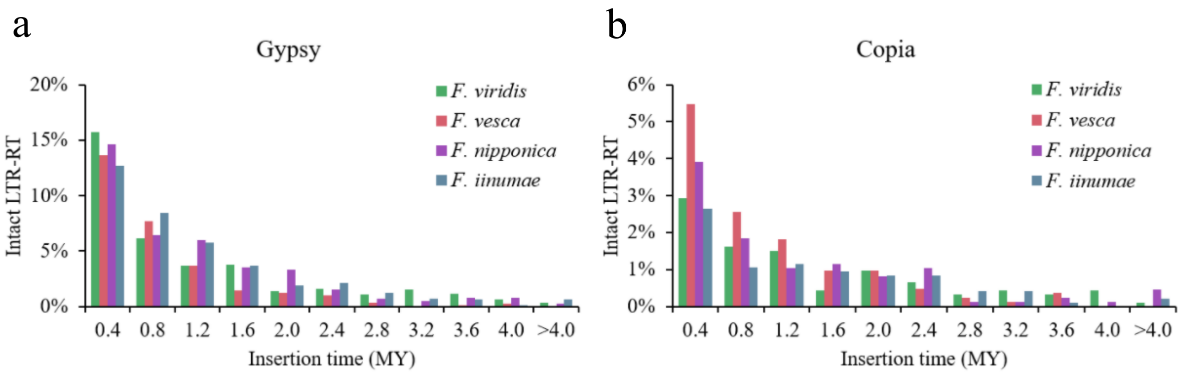

Supplemental Figure 4. Differential fractionation of LTR retrotransposons among subgenomes. **a.** Age distribution of intact LTR-RTs in the Gypsy superfamily among subgenomes. **b.** Age distribution of intact LTR-RTs in the Copia superfamily among subgenomes. Insertion time (MY) was estimated based on mutation rate $\mu = 1.3 \times 10^{-8}$ per bp per year with the Jukes-Cantor adjustment for non-coding sequences. Coloring scheme for the four subgenomes is as described in Figure 1; *F. viridis* (green), *F. vesca* (red), *F. nipponica* (purple) and *F. iinumae* (blue).

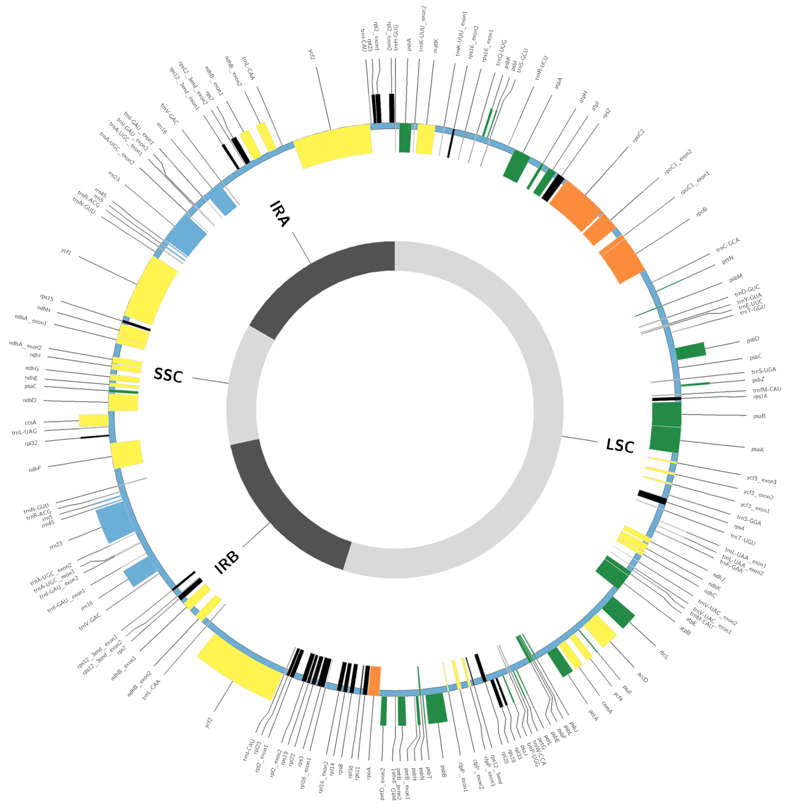

Supplemental Figure 5: The chloroplast genome of 'Camarosa' that was assembled using Verdant [6] and annotated using annoBTD (see URLs). Gene models are shown with various colored boxes on the outer track. The large single copy (LSC) and small single copy (SSC) region and two inverted repeats (IRA and IRB) are shown in the inside track colored light and dark gray, respectively.

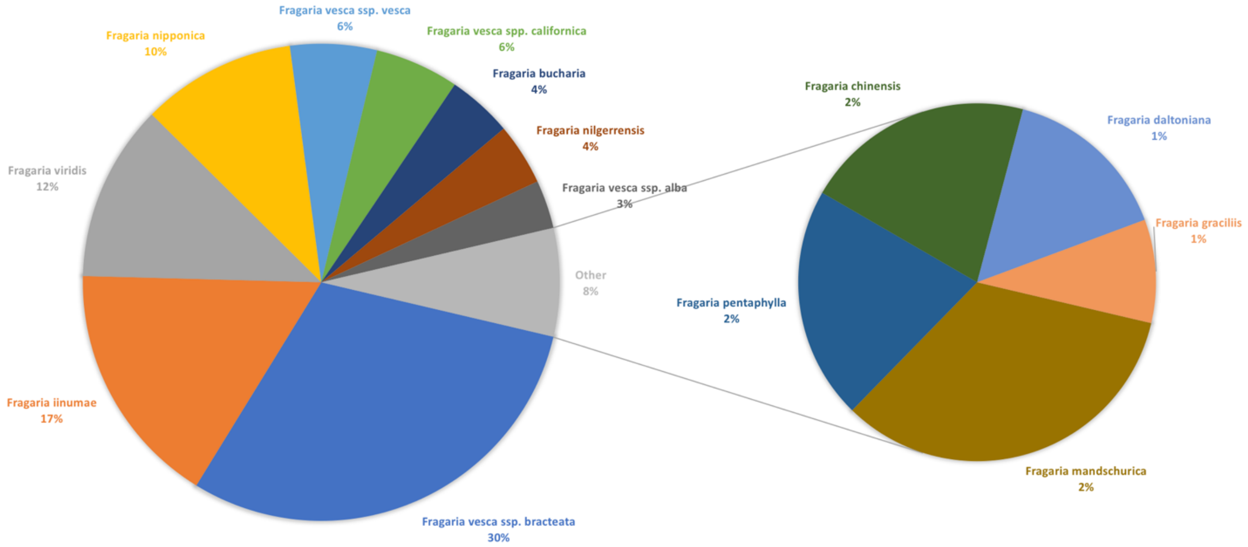

Supplemental Figure 6: PhyDS Output - Proportion of species in a clade with a 'Camarosa' homoeolog. Each species is uniquely colored with matching colored label.

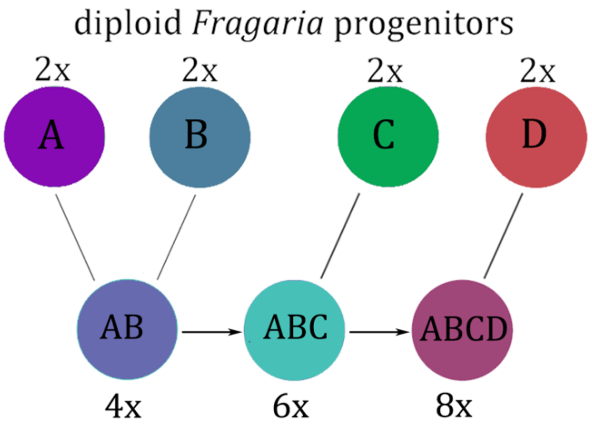

Supplemental Figure 7: Pedigree-type hybridization sequence towards octoploid strawberry - See Figure 2. Species A and B represent *F. nipponica* (purple) and *F. iinumae* (blue), respectively, to form a tetraploid. The ancestral tetraploid strawberry hybridized with *F. viridis* (species C, green) to form the ancestral hexaploid species, which hybridized with *F. vesca ssp. bracteata* (species D, red) to form the octoploid strawberry

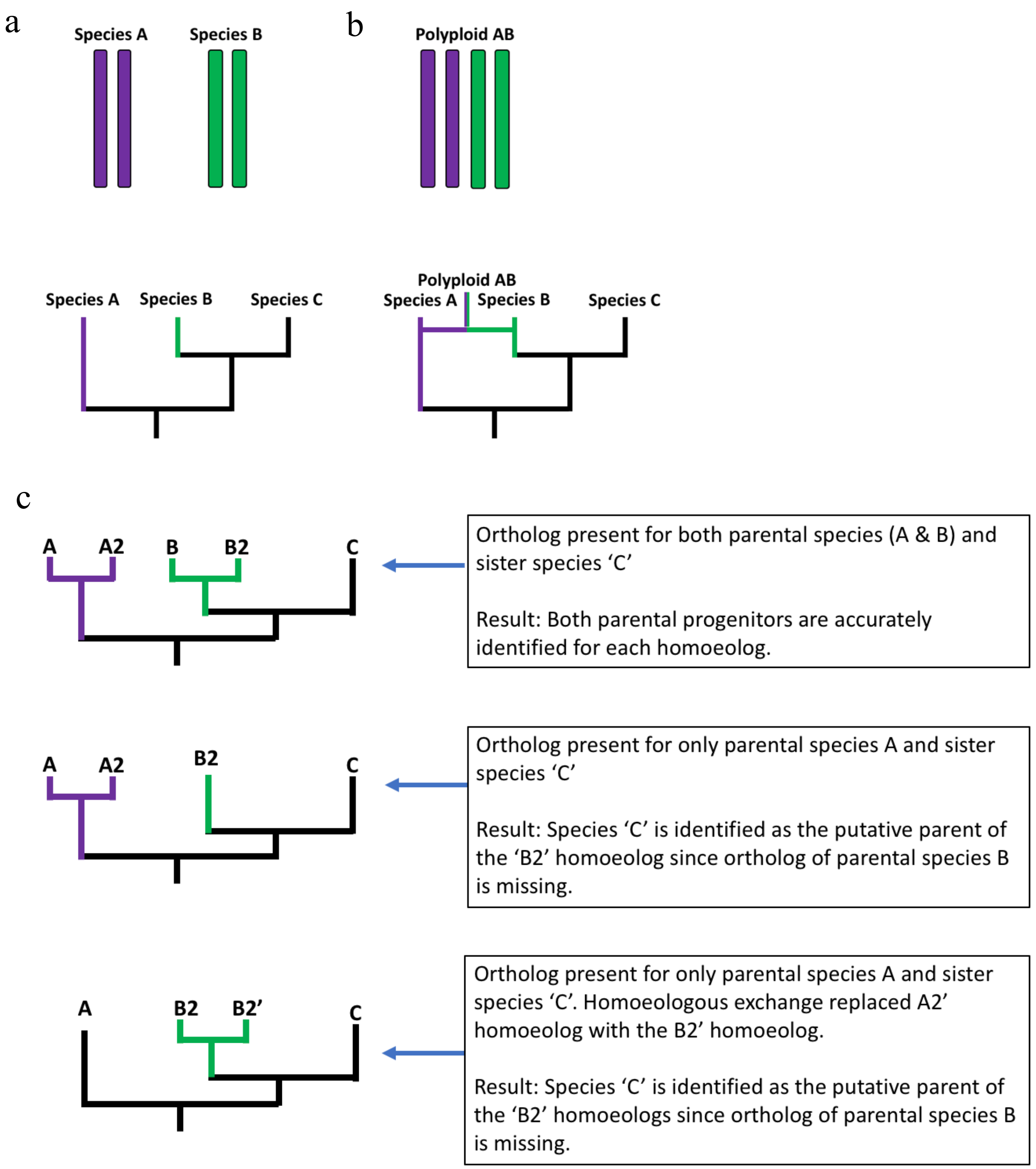

Supplemental Figure 8: Phylogenetic analysis using PhyDS to identify progenitor species of a polyploid event. **a.** Evolutionary relationships among three diploid species, **b.** Species A (purple) and B (green) are involved in an allopolyploidization event, and **c.** Outcomes based on different scenarios that can result in sister species 'C' being incorrectly identified as progenitor due to missing data for species 'B'. This results in related *F. vesca* subspecies being identified as progenitor instead of *F. vesca f. bracteata* (Supplemental Fig. 6).

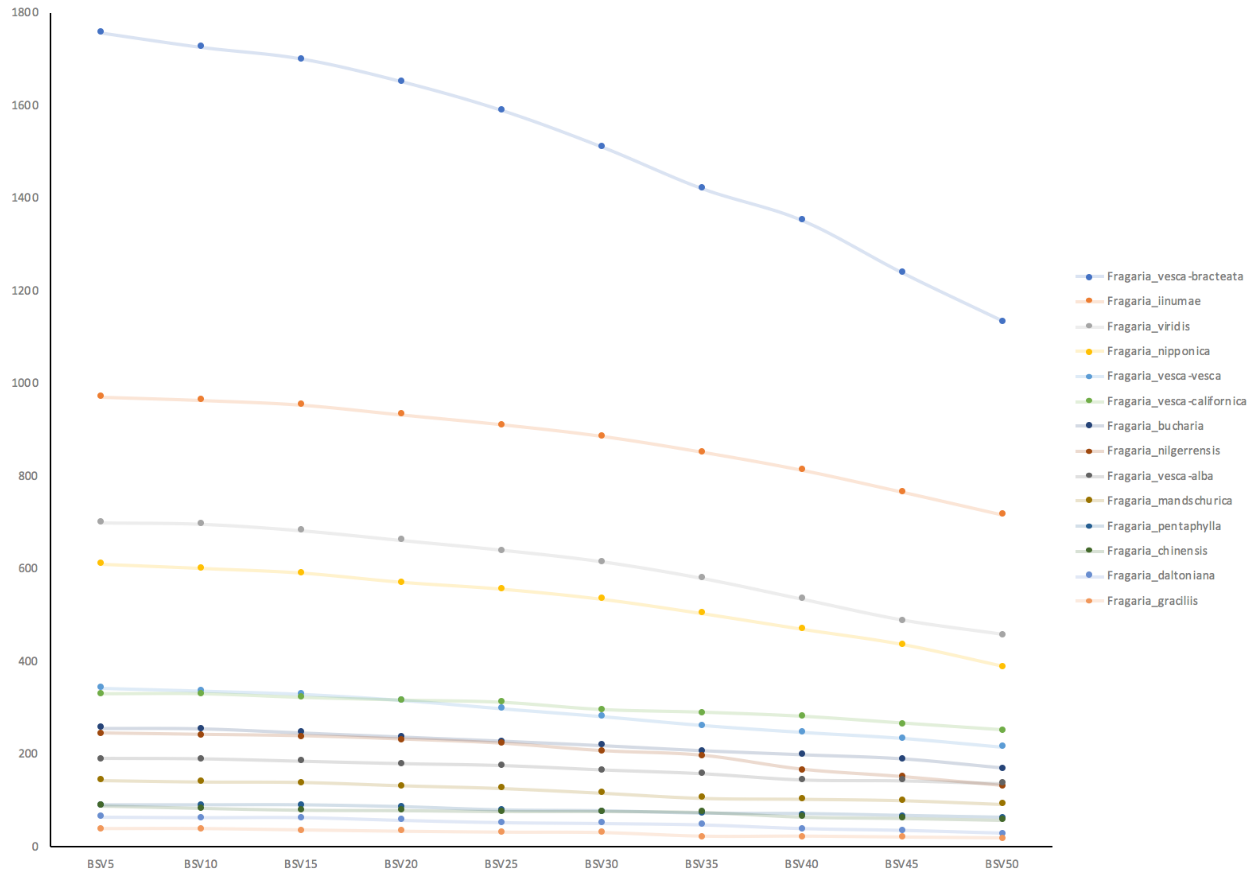

Supplemental Figure 9: The number of orthologous genes (shown on y-axis) from each diploid species that were identified as being sister to a homoeolog from the octoploid genome using PhyDS following various bootstrap support value (BSV) cutoffs (shown on x-axis). The four diploid progenitors were identified as *Fragaria vesca* ssp *bracteata*, *Fragaria iinumae*, *Fragaria viridis*, and *Fragaria nipponica*. Each species is uniquely colored (see figure key).

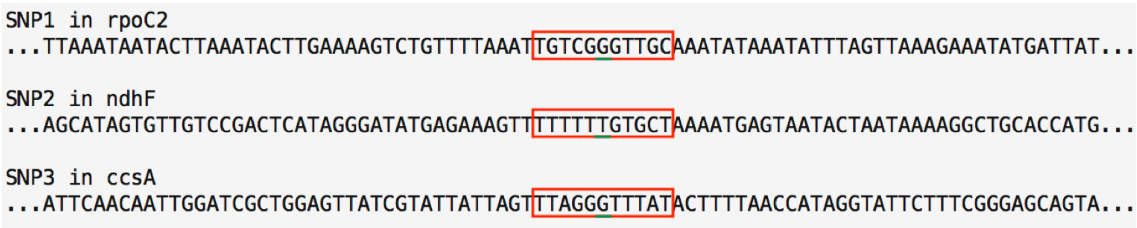

Taxon	Accession	rpoC2 - SNP1	ndhF - SNP2	ccsA -SNP3
F. x ananasas cv. 'Camarosa'	PI 670238	..TGTCGGGTTGC..	..TTTTTTGTGCT..	..TTAGGGTTTAT..
F. vesca f. bracteata	PI 551782	..TGTCGGGTTGC..	..TTTTTTGTGCT..	..TTAGGGTTTAT..
F. vesca f. bracteata	PI 551785	..TGTCGGGTTGC..	..TTTTTTGTGCT..	..TTAGGGTTTAT..
F. vesca f. bracteata	PI 551813	..TGTCGGGTTGC..	..TTTTTTGTGCT..	..TTAGGGTTTAT..
F. vesca f. alba	PI 551841	..TGTCGAGTTGC..	..TTTTTAGTGCT..	..TTAGGATTTAT..
F. vesca subsp. vesca	PI 551649	..TGTCGAGTTGC..	..TTTTTAGTGCT..	..TTAGGATTTAT..
F. vesca subsp. californica	CFRA 2206	..TGTCGAGTTGC..	..TTTTTAGTGCT..	..TTAGGATTTAT..
F. viridis	PI 666621	..TGTCGAGTTGC..	..TTTTTAGTGCT..	..TTAGGATTTAT..

Supplemental Figure 10: A. The regions in the 'Camarosa' genome containing the three parsimony-informative SNPs in the chloroplast genome identified in [7]. B. Sequence alignments of these SNPs in Camarosa, various *F. vesca* subspecies, and a diploid outgroup (*F. viridis*). Camarosa and *F. vesca* f. *bracteata* contain the same SNPs while the other *F. vesca* subspecies and *F. viridis* do not, suggesting plastid donor for the octoploid species was *F. vesca* f. *bracteata*.

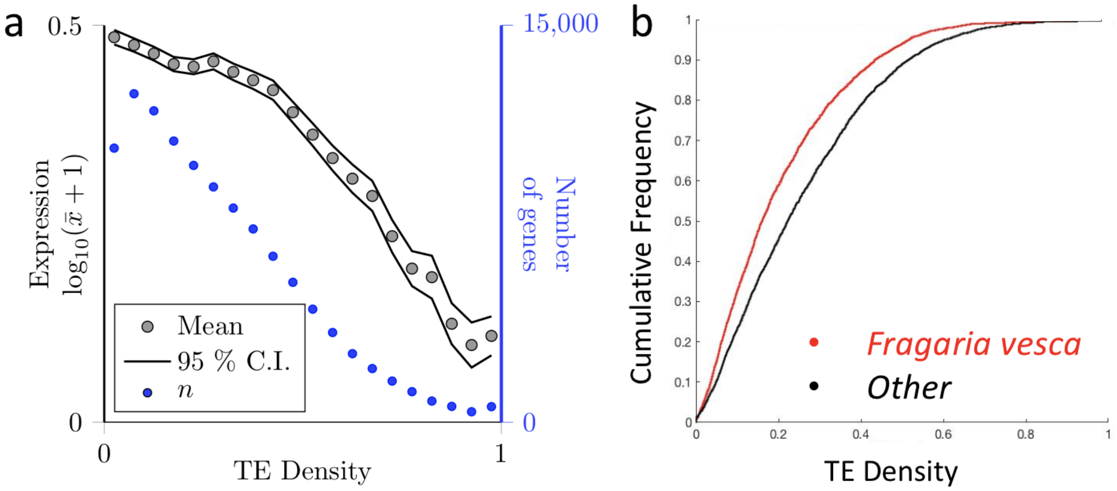

Supplemental Figure 11: Impact of TE density on expression in octoploid strawberry
A. Gene expression is negatively correlated with TE density in octoploid strawberry. TE density was calculated in 5kb up- to 5kb downstream windows of the gene. The total number of genes in each of the twenty bins (5% increments in TE density) is plotted in blue. The mean log-transformed gene expression of each bin with 95% confidence intervals (C.I) is shown. **B.** Comparison of TE densities near genes between the *F. vesca* and average of the three submissive subgenomes. TE densities near genes in the *F. vesca* subgenome are lowest compared to homoeologs in all other subgenomes.

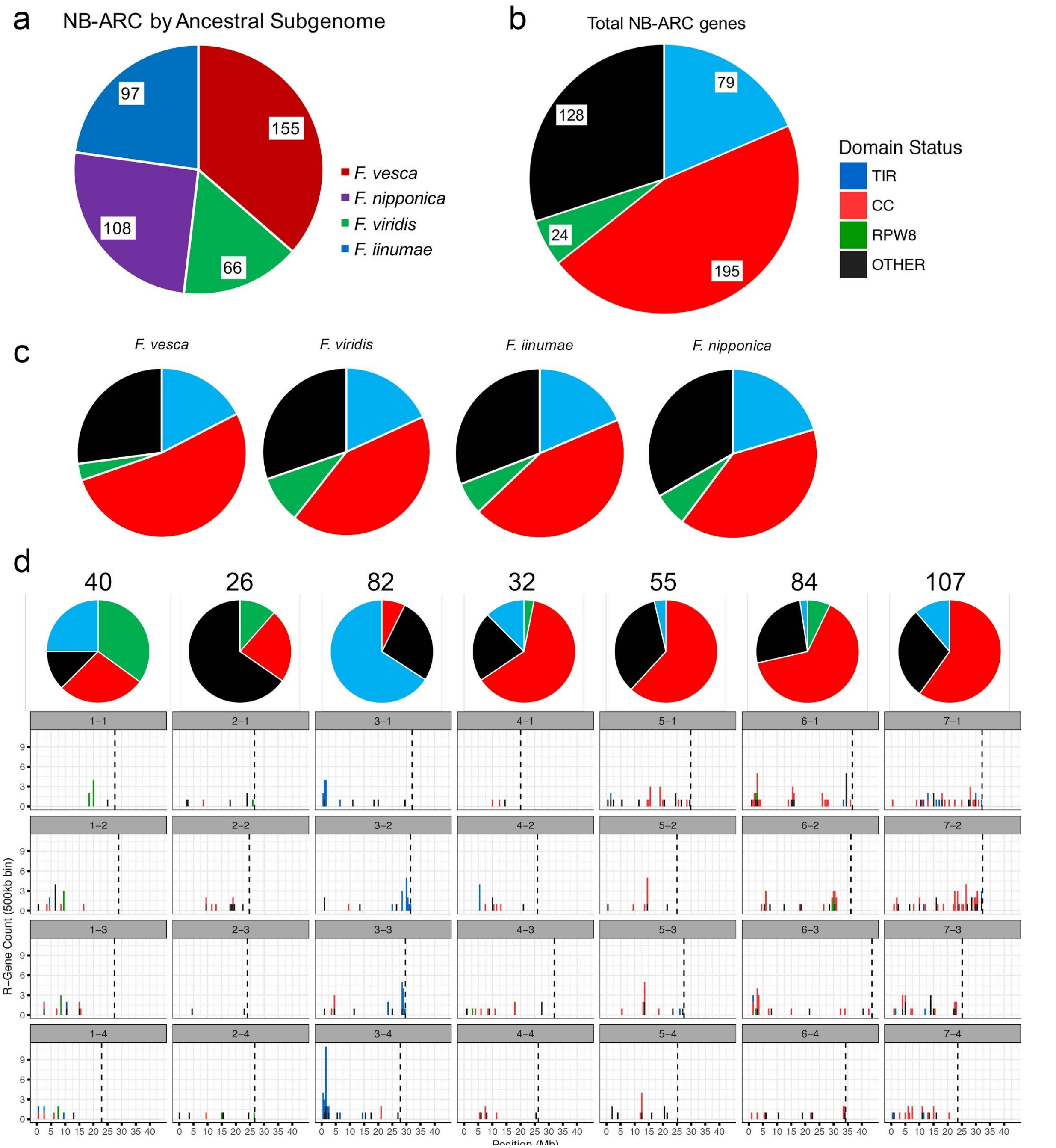

Supplemental Figure 12: Distribution and identities of *R* genes in *F. x ananassa*

A. Total NB-ARC domain-containing *R* genes encoded on respective diploid progenitor genomes; *F. viridis* (green), *F. vesca* (red), *F. nipponica* (purple) and *F. iinumae* (blue). **B.** Proportion of *R* genes encoding CC, TIR, and RPW8 domains. "Other" includes all gene models without one of these domains. **C.** Similar illustration as in B, but for each ancestral genome. **D.** Upper panel, similar analysis as in B, but performed on subsets of *R* genes encoded on homoeologous chromosomes. Lower panel, location of *R* genes on 28 *F. x. ananassa* chromosomes. Y-axis, number of genes; X-axis, position relative to left chromosome end; dashed line, end of chromosome. Bars are colored according to legend in panel B.

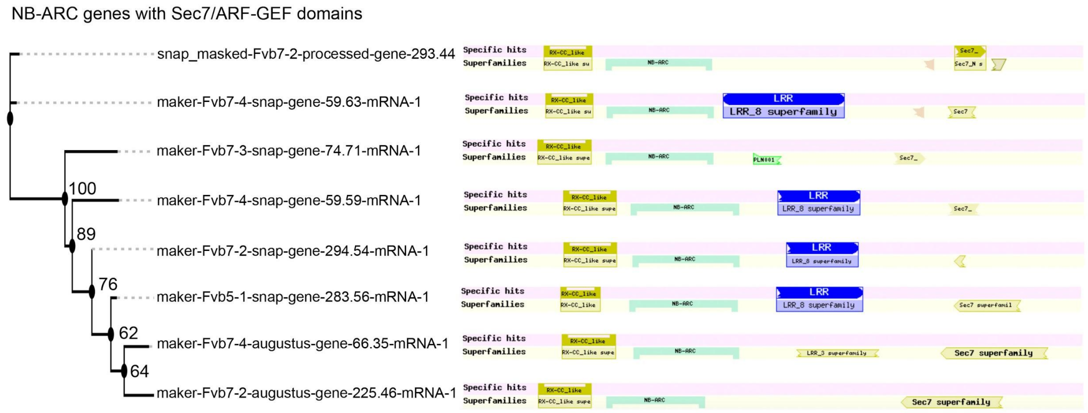

Supplemental Figure 13: R genes encoding Sec7 domains

Domain illustration from the NCBI CD database of eight Fxa proteins with predicted Sec7/ADP-ribosylation factor guanine nucleotide-exchange factor (ARF-GEF) domains. Full length amino acid sequences were aligned by ClustalW and a phylogram with 100 bootstrap supports was generated with FastME 2.0 [8]. ARF-GEFs are a known target of pathogen effectors [9], and this family of *R* genes thus exhibits high potential to function with integrated decoy domains [10].

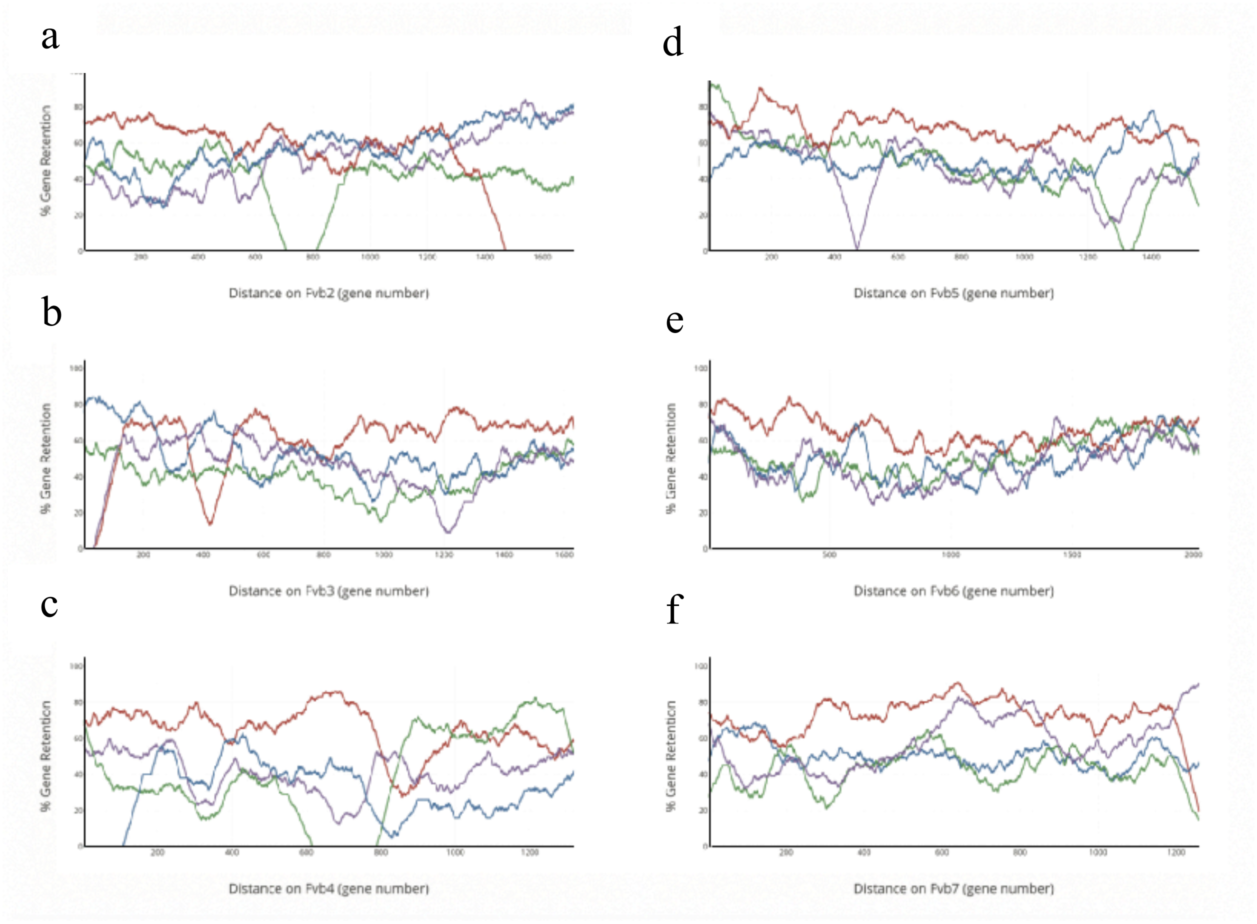

Supplemental Figure 14: Subgenome fractionation patterns
Retention patterns of ancestral genes between the four homoeologous copies of chromosomes 2 through 7 with consistent color coding from Figure 1 for Chromosome 1. *F. viridis* (green), *F. vesca* (red), *F. nipponica* (purple) and *F. iinumae* (blue). The relative distance along the *F. vesca* chromosome is shown on the x-axis with the total number of analyzed genes. The percentage of genes retained is shown on the y-axis estimated using sliding windows of 100 genes. The chromosomes of *Fragaria vesca* V4 are named Fvb1 through Fvb7.

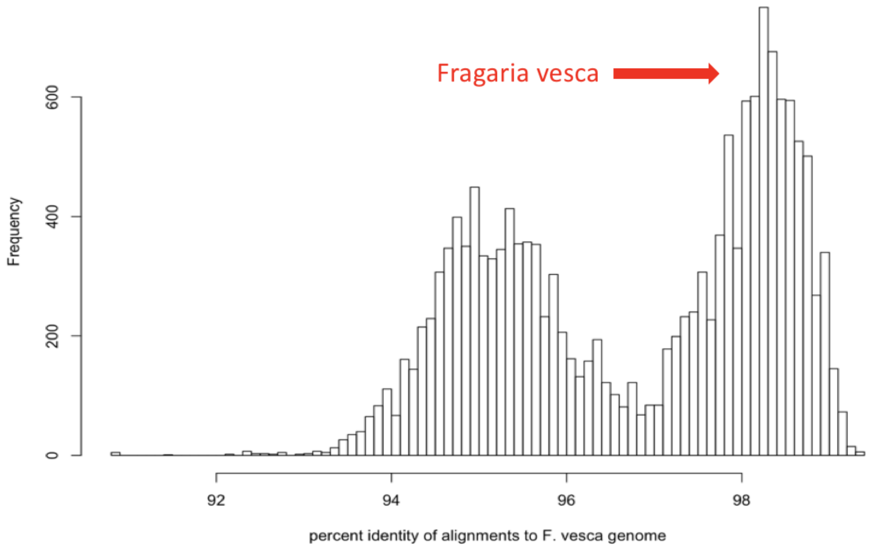

Supplemental Figure 15: Distribution of percent identity of F. x ananassa to F. vesca genes A bimodal distribution can be observed for the 'new world' *F. vesca* var. *bracteata* subgenome 100-97% and the other three subgenomes 93-97% from 'old world' diploid progenitors. These results are supported by phylogenetic analyses.

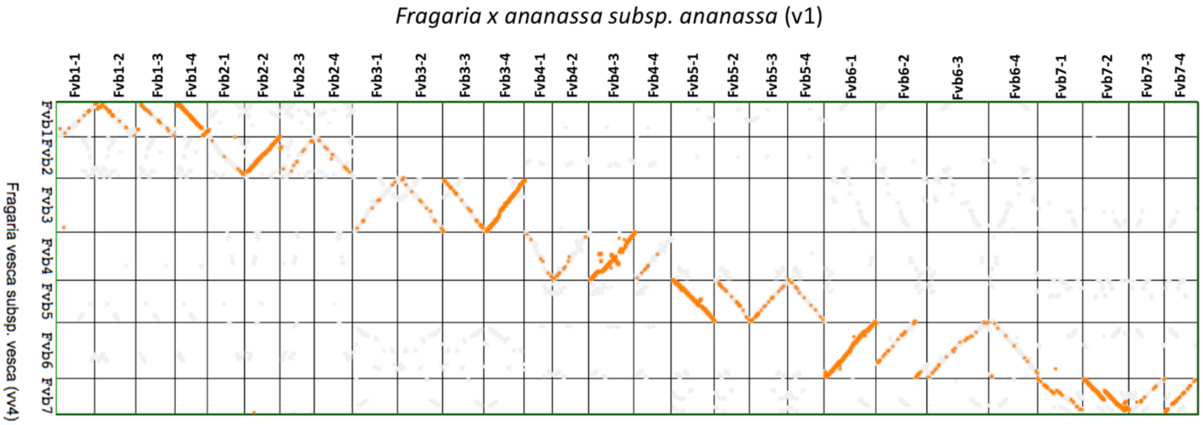

Supplemental Figure 16: Distribution of *F. vesca* subgenome among homoeologous chromosomes The SynMap analysis on CoGe (https://genomevolution.org/coge/) shown above can be regenerated: https://genomevolution.org/r/rvo5 The distribution *F. vesca* subgenome is shown in orange.

Supplemental Tables

Supplementary Table 1: Genomic Sequencing Data

Library type	Reads Length	Insert Size	NCBI SRA #
PCR-free	2x265bp	470	PRJNA508389
PCR-free	2x160bp	800	PRJNA508389
Mate-Pair	2x160bp	3000	PRJNA508389
Mate-Pair	2x160bp	6000	PRJNA508389
Mate-Pair	2x160bp	9000	PRJNA508389
Chromium	2x150bp	-	PRJNA508389

Supplementary Table 2: Assembly Statistics

	Contigs	Scaffolds
**Total Sequences**	48,802	25,426
**Assembly size**	1,206,255,966	1,213,648,575
**Gaps %**	0	0.50
**N50**	79,973	5,980,469
**N50 #sequences**	4,492	64
**N90**	18,617	1,368,136
**N90 #sequences**	15,886	212
**MAX**	616,236	21,879,961

Supplementary Table 3: PacBio Sequencing

Raw reads: total yield (bp)	67,015,644,295
Raw reads: number of reads	5,789,190
Raw reads: mean length (bp)	11,576
Raw reads: N50 read length (bp)	17,699
Error-corrected reads: total yield (bp)	34,825,171,121
Error-corrected reads: number of reads	3,428,345
Error-corrected reads: mean length (bp)	10,158
Error-corrected reads: N50 length (bp)	12,688

Supplementary Table 4: Counts and lengths of annotated features

**Features**	**Protein coding genes**	**lincRNA**	**AOT-lncRNAs**	**SOT-lncRNAs**
Number of genes	108,087	15,621	9,265	5816
Number of exons	582,088	25,687	25,610	16,951
Number of CDS	108,087	-	-	-
Total gene length	341,356,694	9,117,134	13,138,934	10,287,361
Total exon length	169,319,342	7,752,205	4,077,691	5,003,082
Total CDS length	123,648,726	-	-	-
Mean gene length	3,158	585	1,418	1,769
Mean exon length	291	302	159	295
Mean CDS length	1,144	-	-	-

Supplemental Table 5. Summarized BUSCO score of the annotated gene set

Complete BUSCOs	1,385
Complete and single-copy BUSCOs	76
Complete and duplicated BUSCOs	1,309
Fragmented BUSCOs	39
Missing BUSCOs	16
Total BUSCO groups searched	1440

Supplementary Table 6: Transposable elements in the octoploid strawberry genome

	Super-family	No. of TE[a]	Coverage (Mb)	Fraction of genome (%)
**Class I**	LTR/*Copia*	42152	21.64	2.69
	LTR/*Gypsy*	103064	96.17	11.94
	LTR/Unknown	182456	107.88	13.39
	LINE	16686	6.80	0.84
	SINE	239	0.11	0.01
	**Total Class I**	344597	232.60	28.87
**Class II**				
	hAT	19312	7.68	0.95
	MLE	1772	0.31	0.04
	MULE	19507	6.34	0.79
	PIF-Harbinger	4986	1.65	0.20
	Helitron	1526	0.70	0.09
	Maverick	221	0.09	0.01
	Mariner	173	0.4	0.01
	CMC-EnSpm	17984	16.16	2.70
	Unknown	16878	4.50	0.56
	**Total class II**	82359	37.83	5.35
**Total TEs**				
Other unknown repeats		49676	14.95	1.86
**Total Repeats**		301617	216.95	36.08

[a]Intact plus fragmented

Supplemental Table 7: Transcriptome datasets generated in this study for phylogenetic analyses.

CFRA ID	Name	Taxon	Ploidy
1910	F. bucharica	Fragaria bucharica	2
520	F. bucharica	Fragaria bucharica	2
522	F. bucharica 880117 Pakistan	Fragaria bucharica	2
1908	F. chinensis	Fragaria chinensis	2
202	F. chinensis	Fragaria chinensis	2
1685	F. daltoniana #1 China	Fragaria daltoniana	2
1973	F. gracilis	Fragaria gracilis	2
1849	F. iinumae HD-2004-15	Fragaria iinumae	2
1008	F. iinumae Mt. Takeyama (*misidentified, actually F. vesca)	Fragaria iinumae	2
1947	F. mandschurica	Fragaria mandschurica	2
1224	F. nilgerrensis	Fragaria nilgerrensis	2
1610	F. nilgerrensis 96125	Fragaria nilgerrensis	2
1825	F. nilgerrensis Yunnan	Fragaria nilgerrensis	2
2052	F. nipponica seed composite Cluster 10	Fragaria nipponica	2
1863	F. nipponica var. yezoensis HD-2004-72	Fragaria nipponica	2
1797	F. nubicola Nepal 2618	Fragaria nubicola	2
1913	F. pentaphylla	Fragaria pentaphylla	2
1909	F. pentaphylla	Fragaria pentaphylla	2
510	F. vesca f. alba (white)	Fragaria vesca f. alba	2
2095	Hawaii 4 (F7)	Fragaria vesca f. alba	2
429	F. vesca BL-29-1	Fragaria vesca f. bracteata	2
464	F. vesca f. bracteata	Fragaria vesca f. bracteata	2
2178	F. vesca f. bracteata Mary's Peak Conner	Fragaria vesca f. bracteata	2
2177	F. vesca f. bracteata Mary's Peak Road	Fragaria vesca f. bracteata	2
2206	F. vesca californica Strawberry Mountain	Fragaria vesca subsp. californica	2
2219	F. vesca ssp. californica Medicine Creek	Fragaria vesca subsp. californica	2
282	F. vesca subsp. vesca	Fragaria vesca subsp. vesca	2
562	F. vesca subsp. vesca [F. vesca] 89USSR-	Fragaria vesca subsp. vesca	2
1903	F. viridis	Fragaria viridis	2
333	F. viridis	Fragaria viridis	2
2145	F. viridis GE 2012-07	Fragaria viridis	2

.

Chromosome	Progenitor	Chr. Size	PhyDs	Syntelogs
Fvb1-1	F. viridis	27594200	12	1209
Fvb1-2	F. iinumae	28910674	44	1420
Fvb1-3	F. nipponica	27436561	26	1281
Fvb1-4	F. vesca	22887349	108	1768
Fvb2-1	F. nipponica	26582685	15	1583
Fvb2-2	F. vesca	24782128	127	1958
Fvb2-3	F. viridis	24073015	22	1486
Fvb2-4	F. iinumae	26692599	62	1606
Fvb3-1	F. viridis	32005440	32	1587
Fvb3-2	F. iinumae	31459976	63	1555
Fvb3-3	F. nipponica	29626823	25	1349
Fvb3-4	F. vesca	27809139	150	2100
Fvb4-1	F. viridis	20034018	24	1085
Fvb4-2	F. nipponica	25974422	20	1289
Fvb4-3	F. vesca	31955388	121	2442
Fvb4-4	F. iinumae	26295489	44	1227
Fvb5-1	F. vesca	29826953	139	2572
Fvb5-2	F. viridis	24981319	23	1465
Fvb5-3	F. iinumae	27452983	70	1524
Fvb5-4	F. nipponica	25211045	23	1492
Fvb6-1	F. vesca	36657112	159	3189
Fvb6-2	F. nipponica	36124132	34	2065
Fvb6-3	F. iinumae	43627644	71	2400
Fvb6-4	F. viridis	34274104	30	2128
Fvb7-1	F. nipponica	32186896	19	1891
Fvb7-2	F. vesca	32354134	66	2713
Fvb7-3	F. iinumae	25137763	45	1479
Fvb7-4	F. viridis	23534715	17	1352

Supplemental Table 8: Chromosomes shown in Figure 1 with diploid progenitor identified from phylogenetic analysis using PhyDs (Supplemental Figure 6) with assembled chromosome size (length to the basepair is shown). The number of phylogenetically supported genes with bootstrap values >50% identified using PhyDs. The number of syntenic orthologs (syntelogs) with sequence identity as being similar to the "old world" diploid species, shown in Supplemental Figure 15, are provided for the chromosomes identified for *F. viridis*, *F. iinumae*, and *F. nipponica*. The number of syntelogs that are similar to the *F. vesca* genome, shown in Supplemental Figure 15, for *F. vesca* chromosomes are provided.

Supplemental Table 9: Gene and transposable element content per subgenome

Subgenome	Protein Coding Genes	lncRNA Genes	Tandem Duplicates	TE fraction (%)
F. vesca	30922	4674	5458	30.94026772
F. iinumae	27361	4246	4194	38.00048107
F. viridis	23694	3869	3508	38.74786989
F. nipponica	26108	4163	3944	38.61305027
F. vesca	30922	4674	5458	30.94026772
Others Avg	25721	4092.666667	3882	38.45380041
% Diff.	1.202208312	1.142042678	1.405976301	0.804608839

Supplemental Table 10: Summary of Homoeologous Exchanges

Chromosome	Alignment length - NOT F. vesca	Alignment length - F. vesca	Progenitor	F. vesca fraction
Fvb1-2	6,834,277	1,389,655	iinumae	16.9%
Fvb2-4	8,449,988	306,981	iinumae	3.5%
Fvb3-2	9,048,419	467,565	iinumae	4.9%
Fvb4-4	6,563,608	60,514	iinumae	0.9%
Fvb5-3	7,855,363	1,267,336	iinumae	13.9%
Fvb6-3	13,388,952	280,341	iinumae	2.1%
Fvb7-3	7,582,219	378,038	iinumae	4.7%
Fvb1-3	6,394,723	627,886	nipponica	8.9%
Fvb2-1	7,750,305	520,083	nipponica	6.3%
Fvb3-3	7,525,047	1,465,830	nipponica	16.3%
Fvb4-2	6,816,463	129,453	nipponica	1.9%
Fvb5-4	7,110,613	117,436	nipponica	1.6%
Fvb6-2	9,637,646	1,784,054	nipponica	15.6%
Fvb7-1	8,895,463	1,715,314	nipponica	16.2%
Fvb1-4	162,529	12,223,399	vesca	98.7%
Fvb2-2	376,918	13,723,477	vesca	97.3%
Fvb3-4	495,067	15,198,607	vesca	96.8%
Fvb4-3	198,557	17,589,536	vesca	98.9%
Fvb5-1	631,353	17,671,077	vesca	96.6%
Fvb6-1	682,157	22,146,267	vesca	97.0%
Fvb7-2	883,597	18,728,869	vesca	95.5%
Fvb1-1	5,839,084	849,037	viridis	12.7%
Fvb2-3	7,442,520	154,718	viridis	2.0%
Fvb3-1	8,504,313	63,033	viridis	0.7%
Fvb4-1	5,472,382	285,308	viridis	5.0%
Fvb5-2	7,026,077	1,102,193	viridis	13.6%
Fvb6-4	10,310,936	50,881	viridis	0.5%
Fvb7-4	5,710,107	2,259,191	viridis	28.3%

References:

1. Davik, J. *et al.* A ddRAD Based Linkage Map of the Cultivated Strawberry, Fragaria xananassa. *PLoS One* **10,** e0137746 (2015).
2. Edger, P. P. *et al.* Single-molecule sequencing and optical mapping yields an improved genome of woodland strawberry (Fragaria vesca) with chromosome-scale contiguity. *Gigascience* **7,** 1–7 (2018).
3. English, A. C. *et al.* Mind the gap: upgrading genomes with Pacific Biosciences RS long-read sequencing technology. *PLoS One* **7,** e47768 (2012).
4. Walker, B. J. *et al.* Pilon: An Integrated Tool for Comprehensive Microbial Variant Detection and Genome Assembly Improvement. *PLoS One* **9,** (2014).
5. Sargent, D.J. *et al.* HaploSNP affinities and linkage map positions illuminate subgenome composition in the octoploid, cultivated strawberry (Fragaria × ananassa). *Plant Sci.* 242: 140-150 (2016).
6. McKain, M. R., Hartsock, R. H., Wohl, M. M. & Kellogg, E. A. Verdant: automated annotation, alignment and phylogenetic analysis of whole chloroplast genomes. *Bioinformatics* **33,** 130–132 (2017).
7. Njuguna, W., Liston, A., Cronn, R., Ashman, T.L. & Bassil N. Insights into phylogeny, sex function and age of Fragaria based on whole chloroplast genome sequencing. *Mol. Phylogenet. Evol*. **66,** 17-19 (2013).
8. Lefort, V., Desper, R. & Gascuel, O. FastME 2.0: A Comprehensive, Accurate, and Fast Distance-Based Phylogeny Inference Program. *Mol. Biol. Evol.* **32,** 2798–2800 (2015).
9. Nomura, K. *et al.* A bacterial virulence protein suppresses host innate immunity to cause plant disease. *Science* **313,** 220–223 (2006).
10. Kroj, T., Chanclud, E., Michel-Romiti, C., Grand, X. & Morel, J.-B. Integration of decoy domains derived from protein targets of pathogen effectors into plant immune receptors is widespread. *New Phytol.* **210,** 618–626 (2016).
