# Supplementary material for: Origin and evolution of the octoploid strawberry genome
Source: Nat Genet. 2019 Feb 25;51(3):541–7. doi: 10.1038/s41588-019-0356-4 (PMC6882729; doi:10.1038/s41588-019-0356-4)
Supplement: Supplementary file 2 — Reporting Summary [file 41588_2019_356_MOESM2_ESM.pdf]

nature research

Corresponding author(s): Patrick. P. Edger
Steven Knapp

Reporting Summary

Nature Research wishes to improve the reproducibility of the work that we publish. This form provides structure for consistency and transparency in reporting. For further information on Nature Research policies, see Authors & Referees and the Editorial Policy Checklist.

Statistical parameters

When statistical analyses are reported, confirm that the following items are present in the relevant location (e.g. figure legend, table legend, main text, or Methods section).

n/a	Confirmed	
☐	☒	The exact sample size (*n*) for each experimental group/condition, given as a discrete number and unit of measurement
☐	☒	An indication of whether measurements were taken from distinct samples or whether the same sample was measured repeatedly
☐	☒	The statistical test(s) used AND whether they are one- or two-sided<br>*Only common tests should be described solely by name; describe more complex techniques in the Methods section.*
☒	☐	A description of all covariates tested
☐	☒	A description of any assumptions or corrections, such as tests of normality and adjustment for multiple comparisons
☐	☒	A full description of the statistics including central tendency (e.g. means) or other basic estimates (e.g. regression coefficient) AND variation (e.g. standard deviation) or associated estimates of uncertainty (e.g. confidence intervals)
☐	☒	For null hypothesis testing, the test statistic (e.g. *F*, *t*, *r*) with confidence intervals, effect sizes, degrees of freedom and *P* value noted<br>*Give P values as exact values whenever suitable.*
☒	☐	For Bayesian analysis, information on the choice of priors and Markov chain Monte Carlo settings
☒	☐	For hierarchical and complex designs, identification of the appropriate level for tests and full reporting of outcomes
☒	☐	Estimates of effect sizes (e.g. Cohen's *d*, Pearson's *r*), indicating how they were calculated
☐	☒	Clearly defined error bars<br>*State explicitly what error bars represent (e.g. SD, SE, CI)*

Our web collection on statistics for biologists may be useful.

Software and code

Policy information about availability of computer code

Data collection	All commericial DNA and RNA sequencing platforms used in this study are fully described.
Data analysis	All commercial and custom software used in this study for data analysis is fully described including specifying versions used. All custom software developed for this study has already been deposited on Github with weblinks (e.g. Phylogenetic iDentification of Subgenomes; https://github.com/mrmckain/PhyDS).

For manuscripts utilizing custom algorithms or software that are central to the research but not yet described in published literature, software must be made available to editors/reviewers upon request. We strongly encourage code deposition in a community repository (e.g. GitHub). See the Nature Research guidelines for submitting code & software for further information.

Data

Policy information about availability of data

All manuscripts must include a data availability statement. This statement should provide the following information, where applicable:

- Accession codes, unique identifiers, or web links for publicly available datasets
- A list of figures that have associated raw data
- A description of any restrictions on data availability

The genome assembly, annotations, and other supporting data will be made available on Dryad (http://datadryad.org/resource/doi:10.5061/dryad.km0s7k0).

April 2018

Genome assembly and annotation will also be made publicly available on the Genome Database for Rosaceae (https://www.rosaceae.org/) and the CyVerse CoGe platform (https://genomevolution.org/coge/). The raw sequence data will be deposited in the Short Read Archive under NCBI BioProject PRJNA508389. No data restrictions.

Field-specific reporting

Please select the best fit for your research. If you are not sure, read the appropriate sections before making your selection.

☒ Life sciences ☐ Behavioural & social sciences ☐ Ecological, evolutionary & environmental sciences

For a reference copy of the document with all sections, see nature.com/authors/policies/ReportingSummary-flat.pdf

Life sciences study design

All studies must disclose on these points even when the disclosure is negative.

Sample size	A single polyploid genome was sequenced; comparing the expression of all syntenic homoeologous genes across all four subgenomes.
Data exclusions	No data was excluded from any analysis, unless described in the manuscript.
Replication	Flow cytometry measurements were replicated four times. Bootstrapping for phylogenetic analyses were replicated 500 times. Gene expression was compared across all four subgenomes with three distinct tissues to serve as independent replicates.
Randomization	Randomizations were not needed for this study, which involved analyzing subgenomes residing within a nucleus of a single genotype. Plants were grown in a sterile growth chamber.
Blinding	A blinded-experiment is not possible for genome analyses.

Reporting for specific materials, systems and methods

Materials & experimental systems

n/a	Involved in the study
☐	☒ Unique biological materials
☒	☐ Antibodies
☒	☐ Eukaryotic cell lines
☒	☐ Palaeontology
☒	☐ Animals and other organisms
☒	☐ Human research participants

Methods

n/a	Involved in the study
☒	☐ ChIP-seq
☐	☒ Flow cytometry
☒	☐ MRI-based neuroimaging

Unique biological materials

Policy information about availability of materials

Obtaining unique materials	Clones from the sequenced genotype 'Camarosa' are widely available to the community from nurseries around the world. Material is no longer patent protected.

Flow Cytometry

Plots

Confirm that:

☒ The axis labels state the marker and fluorochrome used (e.g. CD4-FITC).

☒ The axis scales are clearly visible. Include numbers along axes only for bottom left plot of group (a 'group' is an analysis of identical markers).

☒ All plots are contour plots with outliers or pseudocolor plots.

☒ A numerical value for number of cells or percentage (with statistics) is provided.

Methodology

Sample preparation	Flow cytometry analyses were conducted by Dr. Arumuganathan in the Flow Cytometry and Imaging Core Laboratory at Virginia Mason Research Center in Seattle, Washington. The procedure used to analyze nuclear DNA content in plant cells was modified from Arumuganathan and Earle (1991). Briefly, the procedure consists of preparing suspensions of intact nuclei by chopping of 50 mg plant plant tissues in MgSO4 buffer mixed with DNA standards and stained with propidium iodide (PI) in a solution containing DNAase-free-RNAase. Fluorescence intensities of the stained nuclei are measured by a flow cytometer. Values for nuclear DNA content are estimated by comparing fluorescence intensities of the nuclei of the test population with those of an appropriate internal DNA standard that is included with the tissue being tested. We use nuclei from Chicken Red blood cells (2.5 pg/2C), Glycine max (2.45 pg. /2C), Oryza sativa cv Nipponbare (0.96 pg/ 2C), Arabidopsis thaliana (0.36 pg/2C) or Zea mays B73 (5.77 pg/2C) as the internal standard. The pellet is suspended by vortexing vigorously in 0.5 mL solution containing 10 mM MgSO4.7H2O, 50mM KCl, 5 mM Hepes, pH 8.0, 3 mM dithiothreitol, 0.1 mg / mL propidium iodide, 1.5 mg / mL DNAse free RNAse (Rhoche, Indionapolis, IN) and 0.25% Triton X-100. The suspended nuclei are withdrawn using a pipettor, filtered through 30-µm nylon mesh, and incubated at 37 °C for 30 min before flow cytometric analysis. Suspensions of sample nuclei is spiked with suspension of standard nuclei (prepared in above solution) and analyzed with a FACScalibur flow cytometer (Becton-Dickinson, San Jose, CA). For each measurement, the propidium iodide fluorescence area signals (FL2-A) from 1000 nuclei are collected and analyzed by CellQuest software (Becton-Dickinson, San Jose, CA) on a Macintosh computer. The mean position of the G0/G1 nuclei peak of the sample and the internal standard are determined by CellQuest software. The mean nuclear DNA content of each plant sample, measured in picograms, are based on 1000 scanned nuclei.
Instrument	FACScalibur flow cytometer (Becton-Dickinson, San Jose, CA)
Software	CellQuest software (Becton-Dickinson, San Jose, CA)
Cell population abundance	The mean nuclear DNA content of each plant sample, measured in picograms, are based on 1000 scanned nuclei.
Gating strategy	The mean position of the G0/G1 nuclei peak of the sample and the internal standard are determined by CellQuest software. See supplemental images.

☒ Tick this box to confirm that a figure exemplifying the gating strategy is provided in the Supplementary Information.
